# A *Pseudomonas aeruginosa*-Derived Particulate Vaccine Protects against *P. aeruginosa* Infection

**DOI:** 10.3390/vaccines9070803

**Published:** 2021-07-20

**Authors:** Zennia Jean C. Gonzaga, Christina Merakou, Antonio DiGiandomenico, Gregory P. Priebe, Bernd H. A. Rehm

**Affiliations:** 1 Centre for Cell Factories and Biopolymers (CCFB), Griffith Institute for Drug Discovery, Griffith University, Don Young Road, Nathan, QLD 4111, Australia; jean.gonzaga@griffithuni.edu.au; 2 Division of Critical Care Medicine, Department of Anesthesiology, Critical Care and Pain Medicine, Boston Children’s Hospital, Boston, MA 02115, USA; christina.merakou@gmail.com (C.M.); gregory.priebe@childrens.harvard.edu (G.P.P.); 3 Department of Anaesthesia, Harvard Medical School, Boston, MA 02115, USA; 4 Discovery Microbiome, Microbial Sciences, Biopharmaceuticals R&D, AstraZeneca, Gaithersburg, MD 34321, USA; antonio.digiandomenico@astrazeneca.com; 5 Division of Infectious Diseases, Department of Pediatrics, Boston Children’s Hospital, Boston, MA 02115, USA; 6 Menzies Health Institute Queensland (MHIQ), Griffith University, Gold Coast, QLD 4222, Australia

**Keywords:** *Pseudomonas aeruginosa*, polyhydroxyalkanoate, vaccine, protective immunity

## Abstract

Despite numerous efforts to develop an effective vaccine against *Pseudomonas aeruginosa*, no vaccine has yet been approved for human use. This study investigates the utility of the *P. aeruginosa* inherently produced polyhydroxyalkanaote (PHA) inclusions and associated host–cell proteins (HCP) as a particulate vaccine platform. We further engineered PHA inclusions to display epitopes derived from the outer membrane proteins OprF/OprI/AlgE (Ag) or the type III secretion system translocator PopB. PHA and engineered PHA beads induced antigen-specific humoral, cell-mediated immune responses, anti-HCP and anti-polysaccharide Psl responses in mice. Antibodies mediated opsonophagocytic killing and serotype-independent protective immunity as shown by 100% survival upon challenge with *P. aeruginosa* in an acute pneumonia murine model. Vaccines were stable at 4 °C for at least one year. Overall, our data suggest that vaccination with subcellular empty PHA beads was sufficient to elicit multiple immune effectors that can prevent *P. aeruginosa* infection.

## 1. Introduction

*P. aeruginosa* is a frequently multiple drug-resistant Gram-negative bacterium that is a ubiquitous opportunistic human pathogen causing life-threatening nosocomial infections, affecting immunocompromised people worldwide [1,2]. This bacterium, which also causes chronic lung infections in people with cystic fibrosis, is extremely adaptive and can survive in a wide range of environments, including different hosts such as plants, animals, and humans [3,4]. This might be due to the complexity and large regulatory network density of the *P. aeruginosa* genome, encompassing 690 genes and 1020 regulatory interactions [3]. In addition, *P. aeruginosa* biofilms can enhance resistance to antibiotic treatment coinciding with emergence of super-resistant cells, which can be responsible for the re-development of the biofilm after antibiotic therapy [4,5,6]. The exopolysaccharide Psl is a key component of *P. aeruginosa* biofilms, and antibodies targeting Psl promote opsonophagocytic killing and are protective in preclinical mouse infection models [7].

Vaccination is likely the best strategy to overcome treatment-associated complications with multi-drug resistance, *P. aeruginosa*. Several vaccines against *P. aeruginosa* have entered clinical trials [8], but none have been approved for human use. The recent Phase II/III clinical trial testing the translational fusion of the OMPs OprF and OprI recombinantly produced by *Escherichia coli* (IC43 vaccine) was unsuccessful to induce the desired immune responses and reduce mortality. These unsuccessful clinical trials strongly indicate a missing link in previous vaccine approaches. *P. aeruginosa* produces several virulence factors and engages complex adaptations to cause infections and resist host immune responses, thereby providing major challenges in vaccine development [3,9,10]. To address the problem, we previously bioengineered *P. aeruginosa* using its inherently assembled polyhydroxyalkanoate (PHA) inclusions as carrier for its own antigens [11]. This approach explored engineering of the *P. aeruginosa* PHA synthase (PhaC1) that catalyzes the polymerization of coenzyme A thioesters of medium chain length 3-hydroxy fatty acids (MCL) to PHA, while PhaC1 remains covalently linked to the surface of the PHA inclusions serving as anchor to display antigens of interest [12]. This was used to engineer translational fusions of PhaC1 with specific antigens mediating the formation of antigen-coated PHA inclusions [11]. Mice vaccinated with isolated PHA inclusions produced a Th1 type immune response characterized by antigen-specific production of IFN-γ and IgG2c isotype antibodies. It should be noted that opsonophagocytic killing activity mediated by serum antibodies in PHA bead-vaccinated groups was significantly greater than in groups vaccinated with soluble antigens, suggesting that PHA beads boosted their immunogenicity. However, to properly evaluate the utility of *P. aeruginosa*-derived antigen-coated PHA beads as the particulate *P. aeruginosa* vaccine requires the further assessment of the functionality of the immune response, such as the analysis of protective immunity by challenging vaccinated animals with the pathogen.

It should be mentioned that most bacteria produce polyhydroxybutyrate (PHB) inclusions as a reserve material. We extensively engineered such PHB inclusions to display pathogen-associated antigens and demonstrated their performance as particulate vaccines in animal models by inducing protective immunity against pathogens, such as *Mycobacterium tuberculosis* [13], *Streptococcus pneumoniae* [14], *Neisseria meningitides* [15] and HCV [16]. No carrier suppression and no adverse effects were detected, while self-adjuvanting [17] and enhanced immunogenicity, when compared to the respective soluble antigens, was observed [18,19].

In this study, we further investigated the utility of the human pathogen *P. aeruginosa* to produce PHA inclusions as innovative vaccines. The PHA bead platform was tested by assessing immunological properties of empty PHA beads and PHA beads engineered to include the vaccine candidate antigens OMPs OprF, OprI, and AlgE as well as a C-terminal fragment of the type III secretion system protein PopB. PopB is a highly conserved protein among *P. aeruginosa* strains and has been previously shown to induce protective Th17 responses in mice after intranasal vaccination when mixed with a Th17 response-eliciting adjuvant called curdlan. This protection was shown to be antibody-independent but IL-17 dependent [20]. Recent attempts to enhance the protective efficacy of the PopB subunit vaccine candidate considered the encapsulation of PopB with its PcrH chaperone into poly-lactic-co-glycolic acid (PLGA) nanoparticles and resulted in the protection of vaccinated mice to acute lethal *P. aeruginosa* pneumonia [21]. The OMPs OprF and OprI are highly conserved, serotype-independent antigens that have shown great promise in several studies [22,23,24,25] although human clinical trials with a hybrid fusion protein of OprF/I failed. The OMP AlgE (alginate pore) is an alternative target that is suggested to be immunogenic and overproduced in the mucoid alginate, overproducing *P. aeruginosa* variants frequently found in the lungs of CF patients [26,27].

Here, *P. aeruginosa* PHA inclusions were engineered for the production of empty PHA beads and PHA beads displaying selected epitopes derived from OMPs OprF/I-AlgE (designated as Ag) or PopB. Respective PHA inclusion fractions were isolated and characterized to evaluate their suitability as particulate vaccines. Protective efficacy of the vaccines after subcutaneous administration was assessed by opsonophagocytic killing assays and survival after a challenge with *P. aeruginosa* in an acute pneumonia model.

## 2. Materials and Methods

### 2.1. Bacterial Strains and Growth Conditions

Bacterial strains, plasmids and primers used in this study are listed in Table S1. *Escherichia coli* XL1-Blue were grown in Luria broth (LB) medium (Thermo Fisher Scientific, Waltham, MA, USA) at 37 °C and was used for plasmid propagation. *P. aeruginosa* strains were grown in LB medium (Thermo Fisher Scientific, Waltham, MA, USA) or mineral salt medium (MSM) [11] at 37 °C for PHA bead production. When required, carbenicillin (Cb; 300 µg/mL) (Invitrogen, Carlsbad, CA, USA) was added. All DNA fragments were purchased from Genscript (Piscataway, NJ, USA). Primers were synthesized by Integrated DNA Technologies (IDT, Coralville, IA, USA).

### 2.2. Plasmid Construction for In Vivo Production of PHA Beads

Cloning techniques were performed as described previously [28]. *P. aeruginosa* codon optimized DNA fragments were used to construct five plasmids in this study (Table S1): (1) pHERD20-T_PopB-PhaC1, (2) pHERD20-T_PopBAg-PhaC1, (3) pHERD20-T_PhaC1-PopB, (4) pHERD20-T_Ag-PhaC1-PopB, and (5) pHERD20-T_PhaC1-PopBAg. Plasmid pHERD20-T_PhaC1-PopB was used for final PHA-PopB bead production and cloning strategy is demonstrated in Figure S1. The DNA fragments (GenScript) were excised by enzyme digestion, followed by fragment separation using agarose gel electrophoresis with SYBR safe stain (Invitrogen) and gel purification (Zymo Research, Irvine, CA, USA). Plasmid isolation was performed using High Pure Plasmid Isolation Kit (Roche, Basel, Switzerland). *Taq* and platinum *pfx* polymerase were purchased from Invitrogen. Final plasmid constructs were confirmed by DNA sequencing (Massey Genome Service, Massey University, New Zealand). Electroporation was used for the transfer of confirmed plasmids into *P. aeruginosa* production strains as previously described [29].

### 2.3. PHA Bead Isolation and Purification

Two *P. aeruginosa* strains were used and compared for PHA bead production: (1) the triple knockout PAO1Δ*phaC1ZC2*Δ*alg8*Δ*pelF* (PAO1ΔCΔ8ΔF) according to Lee et al. 2017 [11] and (2) the quadruple knockout PAO1Δ*phaC1ZC2*Δ*alg8*Δ*pelF*Δ*pslA* (PAO1ΔCΔ8ΔFΔA) (generated from the *P. aeruginosa* PAO1ΔCΔ8ΔA with additional *pslA* gene deletion). *P. aeruginosa* strains were grown under limited nitrogen using sodium gluconate (Thermo Fisher Scientific, Waltham, MA, USA) as a carbon source [12]. MSM media was modified according to Lee et al., 2017 and 300 µg/mL Cb (Invitrogen) was added.

An overnight culture (50 mL) was inoculated from frozen stock and incubated at 37 °C with agitation. The next day, a middle culture (200 mL) was inoculated using 5% (*v/v*) of the overnight culture and was further grown at 37 °C with agitation. On the third day, the main culture (400 mL) was inoculated using the middle culture starting with optical density 600 (OD_600_) of 0.05 and were cultivated at 37 °C with agitation. The cell cultures were induced by 0.5% (*w/v*) arabinose (Thermo Fisher Scientific, Waltham, MA, USA) when OD_600_ reached 0.5–0.8 and incubated further for 48 h at 37 °C with agitation. The cells were harvested by centrifugation at 8000× *g* for 30 min at 4 °C and resuspended in lysis buffer (50 mM Tris, 50 mM Ethylenediaminetetraacetic acid (EDTA), pH 7) (Thermo Fisher Scientific, Waltham, MA, USA) using a homogenizer. The whole-cell lysate was mechanically disrupted using Microfluidizer M-110P (Microfluidics, Westwood, MA, USA) at 20,000 psi. The disrupted cell lysate was centrifuge at 10,000× *g* for 1 h at 4 °C to pellet PHA beads. The isolated PHA beads were washed once with 0.5× lysis buffer (25 mM Tris, 5 mM EDTA, 0.04% (*w/v*) Sodium dodecyl sulfate (SDS), pH 11) (Thermo Fisher Scientific, Waltham, MA, USA) and finally washed with Tris buffered saline (TBS) (50 mM Tris, 150 mM NaCl, pH 7.5) (Thermo Fisher Scientific, Waltham, MA, USA). The purified PHA beads were sterilized with 1 mg/mL Ciprofloxacin (Thermo Fisher Scientific, Waltham, MA, USA) and washed three times with TBS. PHA bead aliquots of 150 µL were plated on LB agar media (Thermo Fisher Scientific, Waltham, MA, USA) and incubated for 24 h. Sterility was achieved when there was no bacterial growth in LB agar plates (Thermo Fisher Scientific, Waltham, MA, USA). The sterile PHA bead vaccines were stored at 4 °C in TBS until formulation for animal trial.

### 2.4. PHA Bead Characterization

The presence of PHA was observed with fluorescence microscopy (FM) by staining the whole cells and the purified PHA beads with lipophilic dye Nile-Red (Thermo Fisher Scientific, Waltham, MA, USA) [30]. Magnafire imaging software was used to capture the images. Transmission electron microscopy (TEM) was used to further confirm the accumulation, shape and size of PHA [31]. PHA beads were processed and sectioned for TEM using 2.5% glutaraldehyde for fixing and 2% agarose for embedding. BioWave processing microwave (PELCO, Fresno, CA, USA) was used for subsequent processing. The TEM images of the sectioned samples were captured using a Hitachi HT7700 (Hitachi, Chiyoda City, Tokyo, Japan) at 80 kV. Gas chromatography–mass spectrometry (GC-MS) was used to analyze 3-hydroxyalkanoate methyl esters of the PHA beads as previously described [32]. The PHA beads (pH 7.5) particle size and zeta-potential were measured using Zetasizer Nano ZS (Malvern Panalytical, Malvern, UK) at room temperature (25 °C). The PHA bead particle size was determined by dynamic light scattering (DLS) analysis. The zeta-potential of the PHA beads was detected by electrophoretic light scattering coupled with phase analysis light scattering. Three technical replicates were performed.

### 2.5. Analysis of the Selected Epitopes/Antigens and HCPs Coating the PHA Beads

Selected fusion proteins along with HCPs displayed on the surface of PHA beads were separated and analysed by sodium dodecyl sulfate-polyacrylamide gel electrophoresis (SDS-PAGE), as previously described [33]. Protein samples were separated in 10% (*vol/vol*) polyacrylamide-separating gels with 4% (*vol/vol*) polyacrylamide-stacking gels at 150 V for 30 min. SDS-PAGE gel was stained by incubating in staining solution (0.05% (*wt/vol*), Coomassie brilliant blue R-250 dye, 50% (*vol/vol*) ethanol, and 10% (*vol/vol*) acetic acid) for 15 min. Subsequently, the gel was destained in 50% (*vol/vol*) ethanol and 10% (*vol/vol*) acetic acid (Sigma, St. Louis, MO, USA) overnight. Images were captured using a Bio-Rad Gel Doc XR+ with the Image Lab Software (Bio-Rad, Hercules, CA, USA). Densitometry was used to analyse protein concentration using bovine serum albumin (BSA) standards (Invitrogen) as described elsewhere [34]. To confirm the identity of the selected proteins and HCPs, Coomassie-stained protein bands of interest were excised and processed for tryptic in-gel digestion as previously described [35]. The resulting tryptic peptide samples were then analyzed by liquid chromatography-tandem mass spectrometry (LC-MS/MS).

### 2.6. Mouse Vaccination and Challenge

(a) Vaccine formulation and immunization. Formulated vaccines contained 10 µg of *P. aeruginosa* antigens/dose of PHA beads, mixed with Alhydrogel^®^ (aluminum hydroxide adjuvant 2%, 25 µL/dose; InvivoGen San Diego, CA, USA) in 200 µL volume incubated for 1 h at room temperature with gentle rotation for adsorption prior to vaccination. Vaccines were mixed with Alhydrogel (InvivoGen) immediately before injection. Animal experiments were approved by the Boston Children’s Hospital IACUC (protocol number 20-12-4326R). FVB/N mice (6–8 weeks old, female) were obtained from the Jackson Laboratories and maintained for both vaccination periods and challenge experiments in the animal facility of Boston Children’s Hospital. Mice were SC vaccinated with Alhydrogel alone (negative control), PHA + Alhydrogel, Ag-PHA + Alhydrogel and PHA-PopB + Alhydrogel suspended in saline. There were 12 mice per group and vaccination was conducted once every two weeks for 6 weeks with a total of 3 vaccinations (Day 0, 14, and 28).

(b) Mouse sera collection. Mouse sera were collected by retro-orbital blood collection on Day 49 of the experiment in serum separator tubes (BD microtainer serum separator tubes). Serum samples were aliquoted and stored at −80 °C until use.

(c) Challenge experiment. For the preparation of the inocula, *P. aeruginosa* strain N13 was plated from a frozen bacterial stock on tryptic soy agar (TSA) and grown overnight at 37 °C. Bacteria were suspended in 10 mL PBS (Invitrogen) until OD_650 nm_ = 0.55. Bacteria we diluted further ¼ in PBS to prepare the inocula. Inocula were verified by serial dilution and plating for CFU enumeration. Mice (8 mice/group) were anesthetized with ketamine/xylazine and intranasal challenge was performed by the administration of 20 μL of the inoculum, applying 10 μL to each nostril (2 × 10^6^ CFU/dose). Mice were monitored for 7 days, and survival percentage was measured. Moribund mice were euthanized.

### 2.7. Serum Antibody Analysis

(a) ELISA. ELISA was performed in order to detect IgG, IgG2 and IgG2c types of antibodies in mouse sera from each vaccine group against Psl [7], PAO1 O-antigen (serotype O2/O5) and T7 LPS, which were kindly provided by G. Pier [36]. High-binding plates (Greiner Bio-One, Frickenhausen, Germany) were coated overnight at 4 °C with 100 µL of 1 µg/mL of the soluble recombinant versions of Ag and PopB/PcrH diluted in PBS (pH 7.5). The next day, the plates were blocked with 200 µL of 5% (*w/v*) skim milk in PBST (PBS with 0.05% (*v/v*) Tween 20; pH 7.5) (Thermo Fisher Scientific, Waltham, MA, USA) for 1 h at 37 °C. Plates were washed three times with PBST and incubated with primary polyclonal antibodies, sera taken from 12 individual mice diluted with 0.5% (*w/v*) skim milk in PBST for 1 h at 37 °C with concentrations ranging from 1:100 to 1:3,276,800 for all types of IgG antibodies tested here, and 1:2 to 1:256 for Psl, O-antigen and LPS antibody analysis. Human anti-Psl mAb (Cam-003 [7]) and rabbit antiserum [37] to PAO1Δ*aroA* were used as positive control for Psl and LPS O-antigen assays, respectively. Plates were washed three times before incubation with the secondary HRP-conjugated antibodies (anti-mouse IgG or IgG1 or IgG2c or anti-rabbit IgG or anti-human IgG) (Abcam, Cambridge, UK) diluted with PBST at a concentration of 1:5000 at 37 °C for 1 h. After washing three times, *o*-phenylenediamine (OPD) (Sigma) was added to plates and incubated for 20 min at room temperature in the dark. The results were measured at 450 nm using BioTek Synergy 2 microplate reader (BioTek, Winooski, VT, USA).

(b) Immunoblot. The specificity of the IgG responses was investigated using immunoblotting analysis. Pooled sera from the vaccinated mice (n = 12) diluted 1:2000 were used against PHA bead after subjected to SDS-PAGE and transfer to nitrocellulose membranes (Life Technology, Waltham, MA, USA). Anti-mouse IgG HRP-conjugate (Abcam) was used as secondary antibody diluted 1:20,000. SuperSignal West Pico Stable Peroxide Solution and SuperSignal West Pico Luminol/Enhancer Solution (Thermo Scientific) were used to develop the signal. The blots were imaged using the Odyssey^®^ Fc Imaging System (LI-COR^®^, Lincoln, NE, USA).

(c) OPK Assay. The OPK assay was performed as previously described, with minor modifications [7]. The assay was done in 96-well plates and had four OPK components (using 0.025 mL each component): (1) *P. aeruginosa* strains from log-phase cultures (~2 × 10^6^ cells/mL dilution); (2) diluted baby rabbit serum (1:10 dilution); (3) differentiated HL-60 cells (2 × 10^7^ cells/mL) as the polymorphonuclear leukocyte source; and (4) monoclonal antibody (mAb) or serum. Luminescent *P. aeruginosa*, constructed as previously described [38], were used with an Envision Multilabel plate reader (PerkinElmer) to determine relative luciferase units (RLUs). The percentage of killing was calculated by comparing the number of colonies or RLUs derived from assays lacking mAb to the number of colonies or RLUs obtained from assays with anti-Psl mAbs or the control mAb.

### 2.8. Statistical Analysis

Analysis of antibody responses was performed using one-way analysis of variance (ANOVA) with statistical significance (*p* < 0.05) indicated by letter-based representation of pairwise comparison between groups using Tukey’s post hoc test. Each data point represents results from 12 mice ± the standard error of the mean.

## 3. Results

### 3.1. Evaluation of the PHA Beads as Particulate Vaccines

**Figure 1:**
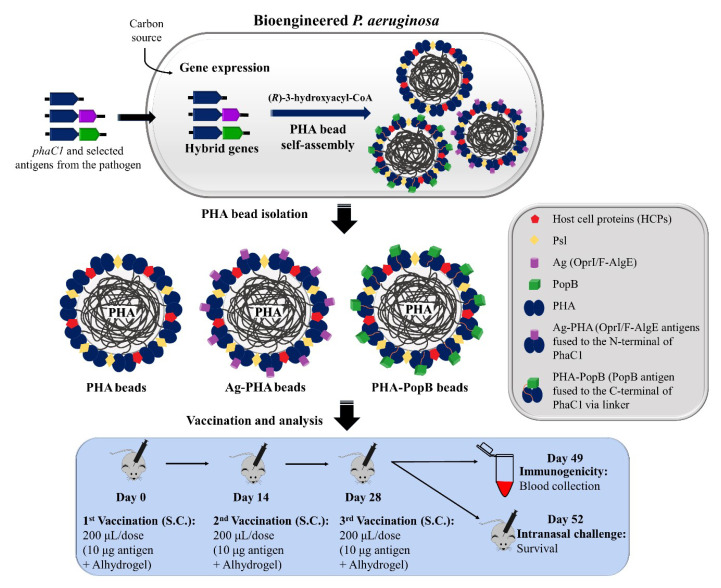
Schematic overview of the production and immunological evaluation of PHA bead vaccines. PHA beads coated with the selected *P. aeruginosa* candidate antigens were produced from *P. aeruginosa* PAO1ΔCΔ8ΔF mutant strain. Plasmid harboring strains were grown under PHA accumulating conditions to mediate overproduction of the fusion protein and subsequent PHA bead self-assembly. Formation of PHA, Ag-PHA and PHA-PopB beads resulted in the display of the selected antigens covalently linked to the PHA synthase along with the HCPs. PHA beads were isolated by mechanical disruption and subsequent purification. Mice were vaccinated with sterile PHA beads via subcutaneous injection with Alhydrogel as an adjuvant. Immunological responses and survival after intranasal challenge with the *P. aeruginosa* strain N13 were measured.

A schematic overview of the production and immunological evaluation of the PHA beads coated with selected antigens produced from *P. aeruginosa* is shown in Figure 1. Bioengineered *P. aeruginosa* overexpressed the integrated hybrid genes *phaC1* (PHA empty bead production), *ag-phaC1* (Ag-PHA bead production) and *phaC1-popB* (PHA-PopB bead production) along with the production of the HCPs. This overexpression, in presence of excess carbon source and nitrogen limiting conditions, mediates assembly of PHA beads densely coated with the respective fusion proteins or empty PHA beads, which can be isolated through mechanical cell disruption. Nitrogen limitation enhances the supply of PHA precursors for efficient PHA synthesis [39]. There is no need for extensive washing because co-purifying HCPs from *P. aeruginosa* are conceived as additional antigens for the induction of a broader immune response. In total, 10 µg of the target fusion protein-based antigens attached to PHA beads or the respective amount of empty PHA beads formulated with Alhydrogel as adjuvant were injected subcutaneously (SC) into FVB/N mice, applying a prime-boost-boost regime. Antibody responses, the opsonophagocytic killing activity of antisera, and protective efficacy against acute lethal *P. aeruginosa* pneumonia were evaluated in mice.

### 3.2. Bioengineering P. aeruginosa for the Production of PHA Bead Vaccines

To develop particulate vaccine candidates for prevention of infection by the highly adaptable human pathogen *P. aeruginosa*, we enhanced the natural formation of PHA beads to serve as particulate vaccines. This was achieved by inactivating competing biosynthesis pathways via gene deletion and by overproducing the PHA synthase. To assess whether PHA beads coated with selected antigens would improve vaccine performance we translational fused antigens to the PHA synthase. In previous studies, PHA inclusions induced dominant Th1 immune responses and the production of functional antibodies facilitating opsonophagocytic killing in mice [11]. HCPs were associated with PHA beads, thereby extending the antigenic repertoire toward a more complex immune response, which we predict is beneficial towards broadening the protective efficacy of vaccination against *P. aeruginosa*. Moreover, not needing to remove HCPs, because they might represent desirable antigens, simplifies purification processes toward more cost-effective vaccine manufacturing. In this study, the PHA bead-based vaccines were shown to be stable at 4 °C for at least 12 months and potentially several years as no significant antigen degradation was observed (Figure S2). In addition, cold temperature for storage may not be necessary [40], making these vaccine materials attractive for uses in developing countries where there might be cold-chain limitations.

In this study, two *P. aeruginosa* strains, (1) the PAO1Δ*phaC1ZC2*Δ*alg8*Δ*pelF* (PAO1ΔCΔ8ΔF) [11] and (2) the PAO1Δ*phaC1ZC2*Δ*alg8*Δ*pelF*Δ*pslA* (PAO1ΔCΔ8ΔFΔA) were tested for the PHA bead and fusion protein production. In the triple knockout *P. aeruginosa* PAO1ΔCΔ8ΔF strain, three key genes (the *phaC1ZC2,* the *alg8* and *pelF*) were deleted in order to improve the PHA bead production process by the inactivation of carbon-flux-competing polymer biosynthesis pathways of alginate and cellulosic pellicle exopolysaccharides. Moreover, these deletions enabled the production of PHA beads exclusively mediated by the introduced PHA synthase and its antigen fusion variants [11,41]. The quadruple knockout mutant *P. aeruginosa* PAO1ΔCΔ8ΔFΔA is a *P. aeruginosa* PAO1ΔCΔ8ΔF derivative with *pslA* gene deletion and a defective Psl polysaccharide production. *P. aeruginosa* PAO1ΔCΔ8ΔF was chosen for further study since this strain enabled the production of all the selected fusion antigens attached to the PHA beads (Figure S3). On the other hand, the *P. aeruginosa* PAO1ΔCΔ8ΔFΔA strain failed to produce detectable amounts of the PhaC1-PopB fusion protein.

**Figure 2:**
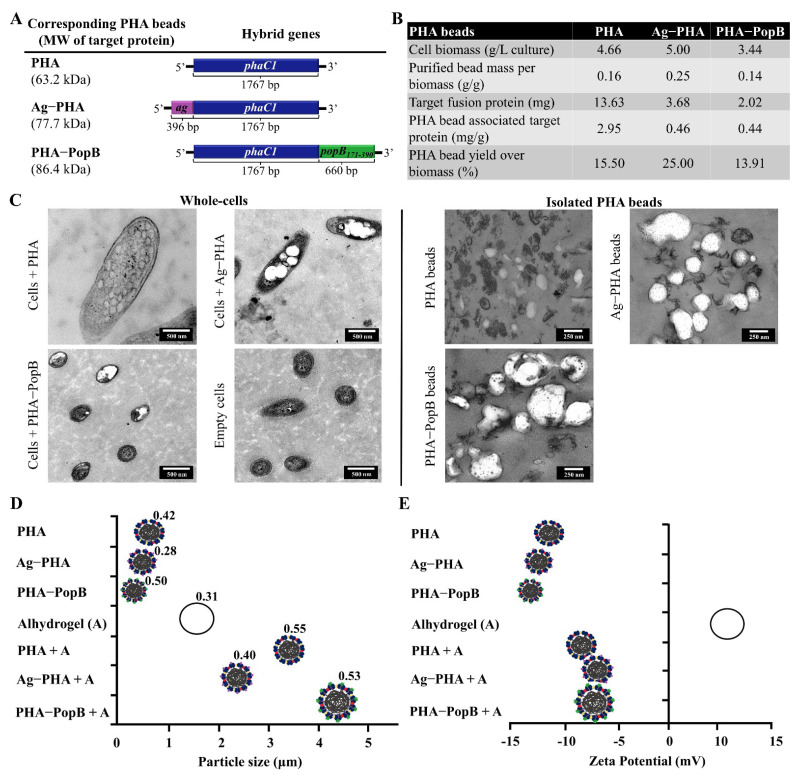
Production and biophysical characterization of PHA beads. (**A**) Schematic representation of hybrid genes encoding fusion proteins for the production of PHA, Ag-PHA and PHA-PopB beads in *P. aeruginosa* PAO1ΔCΔ8ΔF strain. MW (Molecular weight). *phaC1* is the PHA synthase gene. (**B**) Table showing PHA beads composition and yield data. (**C**) TEM images for accumulation and size analysis of PHA beads in whole-cell and isolated PHA beads fractions. (**D**) Size of PHA beads before and after formulation with Alhydrogel adjuvant. Polydispersity indices are shown above the beads. (**E**) Zeta potential of PHA beads before and after formulation with Alhydrogel adjuvant. The particle size and zeta-potential of each PHA beads were measured three times by Zetasizer Nano ZS. Each data point of measurement represents the mean ± SEM. The values are shown in Table S3.

Figure 2 shows the bioengineering of *P. aeruginosa*—production and characterization of the PHA bead vaccines. In this study, three plasmids were used: (1) the pHERD20T-2_PhaC1 (empty PHA bead production); (2) the pHERD20T-2_Ag-PhaC1 (Ag-PHA bead production) [11] and (3) the pHERD20T-2_PhaC1-PopB (PHA-PopB bead production). These three plasmids were transformed into *P. aeruginosa* to enable production of the various particulate vaccines (Figure 2A). Plasmid pHERD20T-2_Ag-PhaC1, mediating the production of PHA beads coated with the OMPs OprF, OprI and AlgE epitopes, was previously described as the most promising vaccine candidate justifying further investigation of vaccine performance in this study [11]. Sequences of OprF, OprI and AlgE are shown in Table S2. To assess alternative vaccine antigens in this platform, a C-terminal fragment of PopB was fused to both the N- and C-termini of PhaC1 but only the C-terminal fusion enabled production of PopB-coated PHA beads. The N-terminal fusion did not mediate any detectable PHA bead formation, which suggests that this fusion most probably rendered PhaC1 synthase inactive. In addition, several attempts were made to incorporate PopB and Ag into one PHA bead, by generating hybrid genes encoding various fusion protein arrangements of PopB (Table S1). However, only the PHA-PopBAg beads were produced, but since the target fusion protein was only detectable by immunoblotting, it was not considered for use in animal experiments (Figure S4). The difficulty in recombinant production of PopB may be due to the absence of its chaperon, PcrH. PcrH has been shown to provide structural support to PopB, leading to its optimal protein formation and production [20,42,43,44].

### 3.3. Biophysical Characterization of PHA Bead Vaccines

PHA bead-producing cells and isolated beads were stained with the lipophilic fluorescent dye Nile red and observed using FM (Figure S5). Fluorescent foci in *P. aeruginosa* cells suggested formation of PHA beads while fluorescence of isolated PHA, Ag-PHA and PHA-PopB beads indicated their identity as beads with lipophilic PHA core. PHA production suggested PhaC1 functionality within the fusion protein was retained. As expected, the negative control *P. aeruginosa* PAO1ΔCΔ8ΔF cells, harbouring an empty plasmid, did not show fluorescence. The table in Figure 2B, indicates the potential cost-effectiveness of the vaccine platform by showing the composition, PHA yield and target fusion protein content of PHA, Ag-PHA and PHA-PopB beads. We used synthetic mineral media free of animal-derived components for PHA bead production, further underpinning the safety of the manufacturing process. It should be noted that strains and production processes were not optimized such as using bioreactors for enhanced upstream processes. Hence, there is the prospect of developing enhanced and more cost-effective manufacturing processes.

The morphology and accumulation of PHA beads were confirmed by TEM (Figure 2C). As previously reported, the spherical shape indicated self-assembly of the PHA beads inside the cell [11,15,31,40]. In addition, the PHA beads appeared to be polydisperse and showed varying beads sizes within the same sample, which is in alignment with previous reports [11,15,33].

To investigate the influence of the Alhydrogel adjuvant used for vaccine formulation, the size and zeta-potential of the PHA beads were measured using Zetasizer Nano ZS before and after the addition of Alhydrogel (Figure 2D,E; Table S3). Before the addition of Alhydrogel, particle sizes of the PHA beads ranged between 0.2–0.6 μm (Table S3) and are polydisperse (>0.1 μm) which is in line with TEM images (Figure 2C). There were no previous reports of the PHA size measurements from *P. aeruginosa*, but our data show resemblance to the size distribution previously described for polyhydroxybutyrate (PHB) beads, the most common PHA [40,45]. The formulation of the PHA beads with Alhydrogel increased the size range to 3.3–4.1 μm mainly due to the size of Alhydrogel itself (1.7 μm) [46]. The increase in size may be due to electrostatic interactions within and between particles or their hybrids or ligand exchange mechanism [46]. In general, antigen presenting cells (APCs) take up particles with a size range of 0.5–10 μm via phagocytosis [47,48]. This allows antigen cross-presentation leading to induction of both humoral and cell-mediate immunity [49]. The negative surface charge of PHA, Ag-PHA and PHA-PopB beads was expected due to the theoretical isoelectric points (pI) of the proteins coating the beads. The theoretical pIs of PHA (PhaC1 anchor protein), Ag-PHA and PHA-PopB are 8.08, 7.68 and 8.78, respectively. Alhydrogel was positively charged. However, although there was a slight shift to positive charge of beads after formulation with Alhydrogel, they remained negatively charged. Surface charge of the particles has been reported to affect cellular uptake—increase in particle uptake by dendritic cells was observed with positive particle surface charge [50]. However, several studies had revealed that APCs are also capable of taking up negatively charged particles due to some cationic sites at the cell membrane facilitating adsorption of the negatively charged particles [51].

### 3.4. Biochemical Characterization of PHA Bead Vaccines

**Figure 3:**
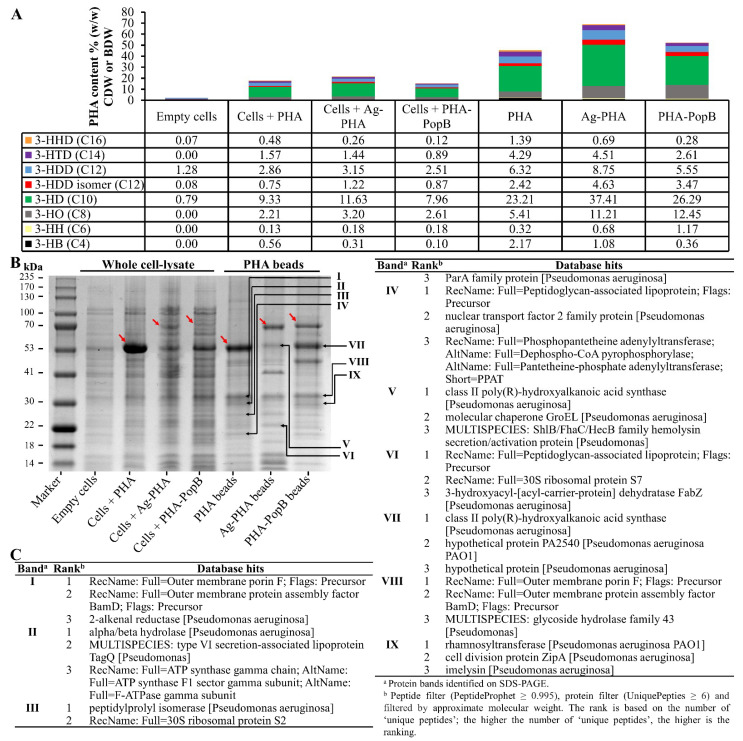
Composition of PHA bead vaccines isolated from engineered *P. aeruginosa*. (**A**) GC-MS for quantification and compositional analysis of PHA beads shown as percentage of CDW or BDW (CDW, cellular dry weight; BDW, bead dry weight). 3-HB (C4), 3-hydroxybutanoate; 3-HH (C6), 3-hydroxyhexanoate; 3-HO (C8), 3-hydroxyoctanoate; 3-HD (C10), 3-hydroxydecanoate; 3-HDD isomer (C12), 3-hydroxydodecanoate isomer; 3-HDD (C12), 3-hydroxydodecanoate; 3-HTD (C14), 3-hydroxytetradecanoate; and 3-HHD (C16), 3-hydroxyhexadecanote. *P. aeruginosa* PAO1ΔCΔ8ΔF is the negative control empty cells. (**B**) Protein profile analysis of whole-cell lysate and the PHA beads separated by SDS-PAGE and gel stained with Coomassie Blue. PHA (63.2 kDa), Ag-PHA (77.7 kDa), and PHA-PopB (86.4 kDa) fusion proteins (in red arrows) along with the HCPs were isolated from *P. aeruginosa* PAO1 ΔCΔ8ΔF strain containing the respective plasmids. Selected co-purifying HCPs were numbered I-IX. The PHA and PHA fusion proteins were confirmed by mass spectrometry (Table S4). (**C**) Table summarizing the identification of the nine PHA bead associated HCPs by MS. Complete list is shown in Table S5.

The composition of PHA bead-producing cells and isolated PHA bead fractions was analysed by GC-MS (Figure 3A). There were very low levels of 3-hydroxyalkanoic acids detected in the negative control *P. aeruginosa* PAO1ΔCΔ8ΔF cells harboring empty plasmid contributing to 2.21% (*w/w*) of cellular dry weight (CDW) and mainly composed of 3-hydroxydecanoate (C10) and 3-hydroxydodecanoate (C12) which is presumably derived from rhamnolipid synthesis [41]. PAO1ΔCΔ8ΔF cells harboring the plasmid mediating formation of empty PHA accumulated PHA contributing to 17.9% of CDW while the strain PAO1ΔCΔ8ΔF containing plasmids mediating Ag-PHA or PHA-PopB bead formation accumulated PHA contributing to 21.4% and 15.2% of CDW, respectively. The isolated PHA, Ag-PHA and PHA-PopB bead fractions had PHA content to approximately 46%, 69% and 52%, respectively (Figure 3A). The isolated PHA beads were mainly composed of 3-hydroxyoctanoate (C8), 3-hydroxydecanoate (C10) and 3-hydroxydodecanoate (C12) and reflected the PHA composition in whole cells. These results were in line with our previous results [11] and reflected the substrate specificity of the PHA synthase of *P. aeruginosa* [52].

The protein profiles of the whole-cell lysates and purified PHA beads were analysed by SDS-PAGE to show the production of full-length fusion proteins and presence of co-purified HCPs (Figure 3B). Dominating protein bands were observed and corresponded to the theoretical molecular weights of PhaC1 (63.2 kDa), Ag-PhaC1 (77.7 kDa) and PhaC1-PopB (86.4 kDa) coating PHA, Ag-PHA and PHA-PopB beads, thus indicating successful bioengineering of *P. aeruginosa* and production of various PHA beads coated with the selected antigens. Densitometry was used to quantify the fusion protein as a percentage of the total protein in PHA bead fractions using BSA standards (Figure S6). Densitometry analysis showed that PhaC1 accounted for 65.7% of the total protein in the PHA bead fraction; Ag-PhaC1 and PhaC1-PopB accounted for 36.4% and 22.4% of the total protein in their corresponding bead fractions, respectively. Protein identity was confirmed using mass spectrometry (MS) for the detection of tryptic peptides (Table S4).

Several additional co-purifying host cell proteins (HCPs) derived from the *P. aeruginosa* were associated with PHA beads, and the major distinct bands were labelled I-IX and selected for identification using MS of tryptic peptides (Figure 3B,C; Table S5). The MS data were filtered according to peptide (PeptideProphet ≥ 0.995) and protein (UniquePeptide ≥ 6) to reveal the identity of the HCPs. Subsequently, the data were filtered based on the apparent molecular weight of the selected proteins. In general, the proposed identities of protein(s) represented by each protein band were ranked according to the number of unique hits revealed during MS and shown in Figure 3C and Table S5 in descending order (the highest number of matching peptide hits has the highest rank). The findings showed that PHA synthase, OprF, peptidoglycan-associated lipoprotein and ribosomal proteins are likely to be consistent co-purifying HCPs, as they were also identified in our previous study [11]. These impurities originating from the pathogen could be beneficial to vaccine efficacy by providing additional antigens/epitopes, and the impurities may also have adjuvant properties towards eliciting improved protective immunity against *P. aeruginosa* infections. Hence, extensive downstream processing may not be necessary to remove the host-cell-derived impurities. However, the process needs to result in consistent antigen composition, as was evidently achieved when comparing these results with those in our previous study [11].

### 3.5. Vaccine Performance of PHA Beads

**Figure 4:**
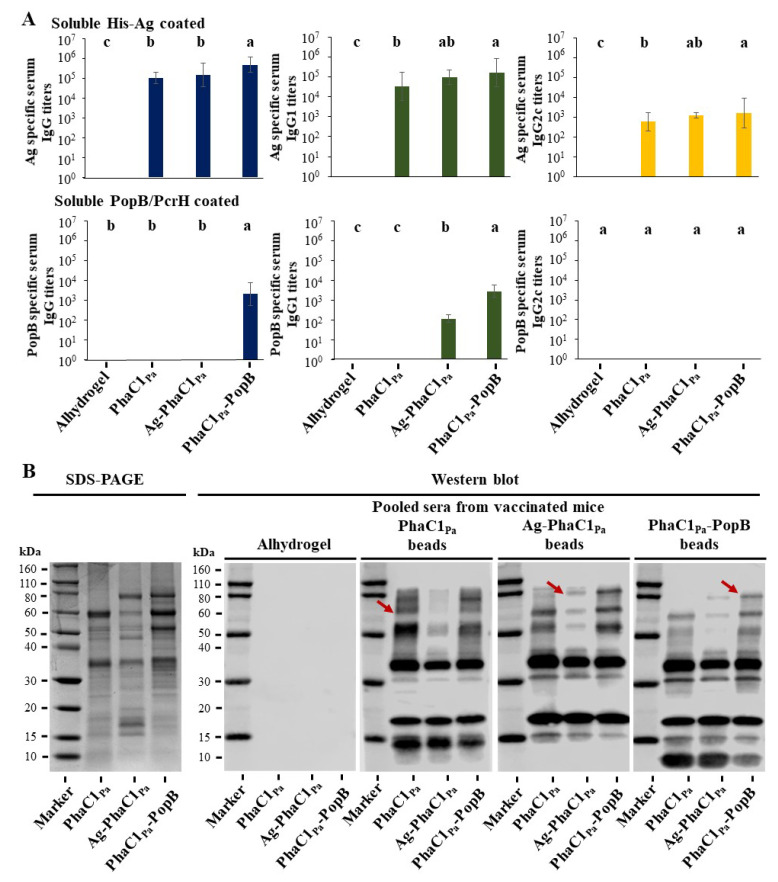
Antigen-specific antibody responses in mice vaccinated with different PHA beads. (**A**) Antigen-specific total IgG, IgG1 or IgG2c antibody responses measured by ELISA. Each data point represents results from 12 mice ± SEM. Statistical analysis was done by one-way ANOVA with statistical significance (*p* < 0.05) indicated by letter-based representation of pairwise comparisons between groups using Tukey’s post hoc test (i.e., data with different letters are statistically significant). (**B**) Specific antigen recognition by immunoblot using pooled antisera from PHA bead-vaccinated mice (n = 12 per group). PhaC1, Ag-PhaC1 and PhaC1-PopB are in red arrows.

The antibody responses of mice vaccinated with PHA beads produced from bioengineered *P. aeruginosa* were analyzed by enzyme-linked immunosorbent assay (ELISA) and immunoblotting (Figure 4). In mice, Th1 immunity is represented by significant induction of IgG2c antibodies, while Th2 immune responses are correlated with IgG1 responses [53]. In this study, total IgG, IgG1 and IgG2c were measured by ELISA to characterize antigen-directed humoral immune responses. Ag (OprF/I-AlgE)-specific total IgG, IgG1 and IgG2c immune responses were detected in all PHA bead vaccinated mice (Figure 4A). The results suggest induction of both Th2 (IgG1) and Th1 (IgG2c) immune responses. Vaccination with PHA and PHA-PopB beads without OprF/I-AlgE antigen also generated Ag-specific IgG, IgG1 and IgG2c antibody responses at similar levels or greater than the Ag-PHA bead group. This indicated an immune response to the co-purified HCPs associated with the empty PHA and PHA-PopB beads. For example, the OprF was identified in bands I and VIII (Figure 3B,C). OprF and OprI are present in high copy numbers in the OM of *P. aeruginosa* [54] while AlgE is barely detectable in the OM of nonmucoid *P. aeruginosa* PAO1 used here for PHA bead production [26]. We assume that during high-pressure microfluidization, OMPs might be released from the OM and attach to the hydrophobic PHA and hence become part of the vaccine formulation. On the other hand, there is no report on the abundance of PopB in the membrane of *P. aeruginosa*. PopB-specific serum IgG titers were detected only in mice vaccinated with PHA-PopB bead but not when vaccinated with empty PHA and Ag-PHA beads suggesting that they are not present as a co-purifying HCP of *P. aeruginosa*. PopB-specific IgG1 titers were found in both the Ag-PHA and the PHA-PopB vaccination group. However, anti-PopB antibody titers were significantly higher in the PHA-PopB vaccination group. IgG2c was not detectable in PHA-PopB vaccinated mice. There was no antibody response in the negative control group vaccinated only with Alhydrogel.

The specificity of the IgGs responses was analyzed by immunoblotting. The reactivity of antibodies in pooled sera from the vaccinated mice was tested against antigens present in the PHA bead formulations (Figure 4B). The results further confirmed that, aside from the selected antigen fusions (PHA, Ag-PHA and PHA-PopB), co-purifying HCPs also contribute to overall immunogenicity of the PHA bead vaccines. The detection of antibodies against a wide range of co-purifying HCPs validated the concept of the large antigenic repertoire using the isolated PHA beads produced by the pathogen. There were no antibodies against PHA bead antigens detected in the pooled sera of the Alhydrogel control group, consistent with the ELISA results. Some of the seroreactive proteins, indicated by red arrows, were also present in the empty PHA bead vaccine and are presumable immunogenic co-purifying HCPs including those exhibiting molecular weights, similar to the target antigens.

ELISA was used to assess anti-Psl polysaccharide antibodies in the sera of mice vaccinated with PHA beads (Figure S7A). The Psl polysaccharide is one type of exopolysaccharide that *P. aeruginosa* produces during biofilm formation [6] and protective monoclonal antibodies targeting this antigen were previously described [7]. An anti-Psl human mAb (Cam-003 [7]) was used here as a positive control. All mice vaccinated with the various PHA beads produced anti-Psl antibodies with similar reactivity, as the positive control mAb, while mice given Alhydrogel alone had no detectable anti-Psl antibodies. This suggests the presence of associated co-purified Psl in all PHA bead formulations. *P. aeruginosa* PAO1 O antigen (O2/O5 serogroup) and T7 lipopolysaccharide (LPS) binding antibodies were also assessed to determine the potential contribution of contaminating *P. aeruginosa* O antigen and/or LPS within the PHA bead formulations (Figure S7B,C). Rabbit antisera to a live-attenuated *P. aeruginosa* strain PAO1Δ*aroA* [37] was used as a positive control. Mice vaccinated with PHA, Ag-PHA and PHA-PopB beads showed low titers of anti-O-antigen and anti-T7 LPS antibodies compared to the positive control, indicating that there might be low amounts of highly immunogenic LPS present in PHA bead formulations and that most antibody responses were due to the selected antigens along with co-purifying HCPs and Psl.

### 3.6. Opsonophagocytic Assay Shows Induction of Functional Antibodies

**Figure 5:**
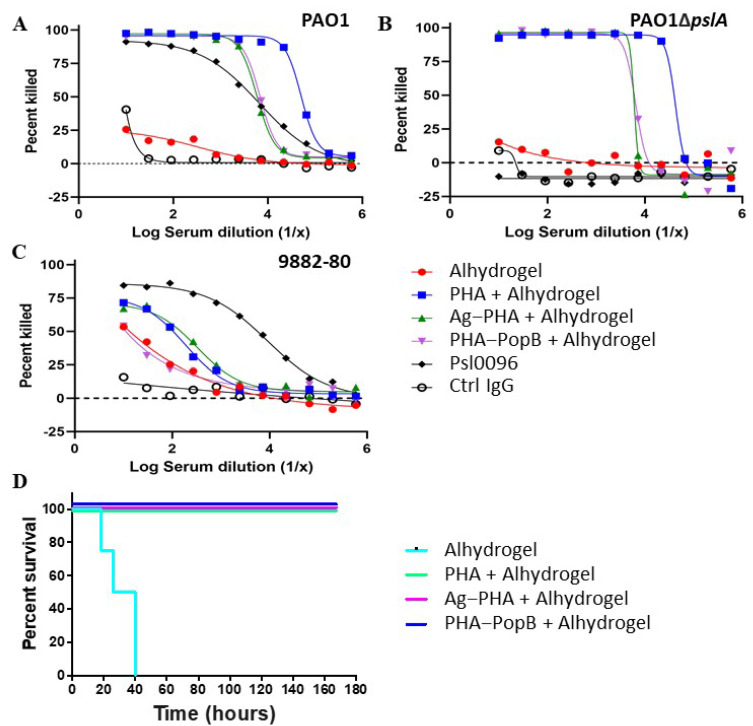
PHA bead vaccines induced functional antibodies and protective immunity. a-c OPK assay against *P. aeruginosa* (**A**) strain PAO1, (**B**) strain PAO1Δ*pslA*, and (**C**) serotype O11 strain 9882-80. Percent killing was calculated relative to results obtained in assays run in the absence of serum/antibody. Results are one representative experiment of three independent experiments performed. (**D**) Survival of the vaccinated FVB/N mice after challenge with the serotype O6 *P. aeruginosa* strain N13. Mice (n = 12 per group) were immunized SC with Alhydrogel, PHA bead + Alhydrogel, Ag-PHA bead + Alhydrogel, or PHA-PopB bead + Alhydrogel, once every 2 weeks for 6 weeks (3 doses), and then intranasally challenged 3 weeks later.

To assess the functionality of PHA bead-induced antibody responses, we performed opsonophagocytic killing (OPK) assays using *P. aeruginosa* strains PAO1 (a wild-type serogroup O2/O5 non-cytotoxic strain) [55], PAO1Δ*pslA* (Psl-negative strain), 9882-80 (serogroup O11) [7,56] as targets (Figure 5A–C). Antisera from mice immunized SC with PHA, Ag-PHA, and PHA-PopB beads (plus Alhydrogel) had significantly greater killing activity against both the PAO1 and PAO1Δ*pslA* strains when compared to the Alhydrogel control group. A lower percentage killing was observed when the *P. aeruginosa* strain 9882-80 was used. Overall, the opsonophagocytic killing assay data suggest that all PHA bead formulations were capable of inducing functional antibodies mediating OPK activity against the three tested *P. aeruginosa* strains. These data also indicated an induction of a serotype-independent antibody response.

### 3.7. PHA Bead Vaccine Formulations Induce Protective Immunity

In order to assess the protective efficacy of the different PHA bead vaccine formulations, we used a murine acute pneumonia model [20]. Figure 5D shows that all mice vaccinated with the various PHA bead formulation plus Alhydrogel survived the intranasal challenge with *P. aeruginosa* strain N13, a wild-type serotype O6 early clinical isolate from a CF patient [55,57]. All mice from the Alhydrogel control group died within 40 h after the start of the challenge. Although antigen/epitopes were not engineered into the surface of the empty PHA beads, the presence of associated OMPs, other HCPs and Psl may have contributed to the induction of opsonizing antibodies mediating 100% survival in the challenge experiment. Overall, these data suggest that PHA bead formulations produced in this study induce serotype-independent protective immunity against *P. aeruginosa* infection.

## 4. Discussion

This study utilized the inherent PHA synthesis capability of *P. aeruginosa* to manufacture its own empty PHA beads and PHA beads coated with selected antigens as particulate vaccine candidates. *P. aeruginosa* is one of the many bacteria that produce PHA inclusions for carbon and energy storage [58]. Vaccine candidate antigens/epitopes were engineered to coat the PHA beads during in vivo assembly and a downstream process was devised to retain associated HCPs as well as the polysaccharide Psl, another candidate vaccine antigen. These PHA bead fractions contained a repertoire of antigens that might contribute to induction of a complex immune response, which could be required to protect against highly adaptable *P. aeruginosa*. Extensive downstream processing to remove host cell derived impurities (i.e., HCPs) was not required as impurities were deemed to be beneficial additional antigens boosting vaccine performance. A less stringent downstream process combined with the yield achieved in this study are suggesting a cost-effective manufacturing process underlying the tested PHA bead vaccine candidates (Figure 2). Purified antigen-coated PHB beads produced by recombinant *E. coli*, in the context of other bacterial and viral pathogens, were previously shown to exhibit superior immunological properties, when compared to the respective soluble antigens [14,15,16,33,59,60,61]. Characterization of our *P. aeruginosa* PHA beads showed that they exhibit a size range (0.2 to 0.6 μm without Alhydrogel adjuvant; 2.2 to 4.2 μm with Alhydrogel adjuvant) suitable for efficient uptake by APCs [48,49] (Figure 2). PHA beads comprised a significant fraction of the target vaccine candidate antigens and co-purifying mostly cell envelope associated antigens (HCPs) that were conceived to broaden the immune response (Figure 3). Indeed, an immunogenicity study confirmed that the target antigens and the HCPs were immunogenic as demonstrated by immunoblotting (Figure 1 and Figure 4). All PHA bead formulations, including the empty PHA beads not engineered to display target antigens induced antibodies against the epitopes derived from OMPs OprF, OprI and AlgE suggesting that these OMPs are present as HCPs as further confirmed by MS analysis (Figure 3 and Figure 4). These antibodies contained a significant fraction of IgG2c isotypes indicative of an induced Th1 mode of immune response, i.e., induction of cell-mediated immunity. Since anti-PopB antibodies were predominantly found in the group vaccinated with PopB-coated PHA beads, PopB does not represent a part of the HCP fraction as corroborated by MS analysis of PHA bead associated HCPs (Figure 3). All vaccines induced strong IgG1 responses indicative of induction a Th2-type immune response. Serum antibodies were functional, mediating specific and serotype-independent OPK activity directed against different *P. aeruginosa* strains (Figure 5).

PHA bead formulations induced protective immunity in a murine acute pneumonia model of *P. aeruginosa* infection. Since the *P. aeruginosa* strain N13 (serogroup O6) used for infection differed in serogroup from the vaccine production strain PAO1 (serogroup O5) and the OPK activity was serotype-independent, LPS is likely not contributing to the overall immunity. This was corroborated by a lack of anti-LPS responses (Figure S7). However, a significant anti-Psl antibody response suggested that Psl was co-purified. We deliberately did not knockout Psl biosynthesis in our production strain to enable that Psl further expand the antigenic repertoire of our PHA bead formulations [7]. Although, the OPK assay showed efficient killing of *P. aeruginosa* PAO1ΔpslA (Figure 5), anti-Psl antibody responses possibly contribute to protective immunity. The expanded antigen repertoire in PHA bead formulations might have crucially contributed to induction of a complex immune response mediating strong protective immunity.

Some studies had suggested that a Th17 response may be the missing piece essential for an effective *P. aeruginosa* vaccine as it plays a key role in innate and adaptive antibacterial defense [20,62,63,64,65]. Although the Th17 immune response was not assessed in this study, PopB is a known stimulator of Th17 immunity. However, without PopB, PHA and Ag-PHA bead vaccinated mice were fully protected against *P. aeruginosa* infection (Figure 5D). It should be noted that in the OPK assay with *P. aeruginosa* strain 9882-80, PHA-PopB bead vaccines induced no significant levels of opsonizing antibodies. This suggested that PopB-mediated Th17 immunity might not be required for the induction of protective immunity in this type of vaccine. The overall vaccine efficacy might be due to the combination of antigens inducing a mixed Th1- and Th2-associated antibody response, i.e., combined induction of functional opsonizing antibodies and cell-mediated immunity [11]. Several studies had shown the importance of humoral immune response in protection against *P. aeruginosa* [53,66], while the requirement for cell-mediate immune responses is less well established.

Several vaccine candidates were developed and tested in clinical trials but still no *P. aeruginosa* vaccine has been approved. Despite an abundance of research, it remains unclear what kind of immune response is required for an effective *P. aeruginosa* vaccine. Early vaccine candidate research focused on the LPS O antigen, which induced high levels of immunity, but this protection was serotype dependent not accounting for the more than 30 subtypes of LPS antigen [67,68]. More recent studies suggested that a combination of cellular and humoral immunity is needed to provide protection. Studies have shown a Th1-dominated immunity, independent of production of antibody, provided protection during chronic *P. aeruginosa* pneumonia [69,70,71]. Another study described the protection of PopB-immunized mice from lethal pneumonia in an antibody-independent but Th17-mediated immune response [20]. Growing evidence suggests that Th17 play an important role in innate and adaptive host immunity, and in autoimmunity [21,62,63,64,65,72]. More recent studies reported that Th2-predominant responses resulted in bacterial load reduction and protection [71,73]. Furthermore, other immune mechanisms have been implicated to provide protection against *P. aeruginosa* infection, such as Th22 cells [74], macrophages [74], and neutrophils [55].

## 5. Conclusions

PHA beads induced functional antibodies leading to opsonophagocytic killing and an overall serotype-independent immune response that protected mice against acute infection by *P. aeruginosa*. The PHA bead-associated HCPs and Psl likely played an important role in broadening the immune response toward protective immunity as supported by the performance of empty PHA bead fractions to induce antibodies mediating OPK and protective immunity. The antigens/epitopes engineered into the PHA bead surface were not required to induce protective immunity. The PHA bead fractions produced by bioengineered *P. aeruginosa* were consistent, safe, well tolerated and showed a shelf-life of >12 months at 4 °C. This PHA bead vaccine platform offers an attractive new strategy toward the development of vaccines against this challenging pathogen *P. aeruginosa*. Moreover, this concept might also be applied to other pathogens that are inherently capable of producing PHA inclusions, such as *Acinetobacter baumannii*, *Burkholderia* spp., *Bacillus anthracis, Vibrio cholerae, Bordetella pertussis, Rickettsia* spp., *Legionella* spp. and *Mycobacterium tuberculosis*.

## Data Availability

The data are available under reasonable request to the corresponding authors.

## Supplementary Materials

The following are available online at https://www.mdpi.com/article/10.3390/vaccines9070803/s1, Table S1: Bacterial strains, plasmids and primers used in this study; Figure S1: Strategy for the construction of pHERD20T-2_PhaC1-PopB for PHA-PopB bead vaccine production; Figure S2: Stability study; Figure S3: Target fusion protein production in *P. aeruginosa* PAO1ΔCΔ8ΔF and PAO1ΔCΔ8ΔFΔA production strains containing the respective plasmids for the production of PHA, Ag-PHA and PHA-PopB, respectively; Table S2: Sequences of OprF, OprI and AlgE; Figure S4: Immunoblot for detection of target fusion proteins using anti-PHA synthase antibodies; Table S3: Particle size and zeta-potential measurements of the PHA bead vaccines; Figure S5: Fluorescence microscopy images of whole-cells and PHA beads produced in bioengineered *P. aeruginosa* PAO1ΔCΔ8ΔF harbouring various fusion protein-encoding pHERD20T-2 expression vectors stained with Nile-red; Figure S6: Protein quantification of the purified PHA bead vaccines by densitometry; Table S4: Mass spectrometry (MS) analysis for identification of PHA synthase and PHA synthase fusion proteins; Table S5: Identification of the nine PHA bead associated HCPs by mass spectrometry; Figure S7: Anti-Psl and anti-LPS O-antigen polysaccharide responses in mice vaccinated with various PHA beads.

## References

[1] Sharma A., Krause A., Worgall S. (2011). Recent developments for Pseudomonas vaccines. Hum. Vaccines.

[2] Grimwood K., Kyd J.M., Owen S.J., Massa H.M., Cripps A.W. (2015). Vaccination against respiratory Pseudomonas aeruginosa infection. Hum. Vacc. Immunother..

[3] Moradali M.F., Ghods S., Rehm B.H.A. (2017). Pseudomonas aeruginosa Lifestyle: A Paradigm for Adaptation, Survival, and Persistence. Front. Cell. Infect. Microbiol..

[4] Thi M.T.T., Wibowo D., Rehm B.H.A. (2020). Pseudomonas aeruginosa Biofilms. Int. J. Mol. Sci..

[5] Brooun A., Liu S., Lewis K. (2000). A Dose-Response Study of Antibiotic Resistance in Pseudomonas aeruginosa Biofilms. Antimicrob. Agents Chemother..

[6] Ghafoor A., Hay I., Rehm B.H.A. (2011). Role of Exopolysaccharides in Pseudomonas aeruginosa Biofilm Formation and Architecture. Appl. Environ. Microbiol..

[7] Di Giandomenico A., Warrener P., Hamilton M., Guillard S., Ravn P., Minter R., Camara M.M., Venkatraman V., MacGill R.S., Lin J. (2012). Identification of broadly protective human antibodies to Pseudomonas aeruginosa exopolysaccharide Psl by phenotypic screening. J. Exp. Med..

[8] Merakou C., Schaefers M., Priebe G.P. (2018). Progress Toward the ElusivePseudomonas aeruginosaVaccine. Surg. Infect..

[9] Gellatly S.L., Hancock R.E.W. (2013). Pseudomonas aeruginosa: New insights into pathogenesis and host defenses. Pathog. Dis..

[10] Priebe G.P., Goldberg J.B. (2014). Vaccines for *Pseudomonas aeruginosa*: A long and winding road. Expert Rev. Vaccines.

[11] Lee J.W., Parlane N.A., Wedlock N., Rehm B.H.A. (2017). Bioengineering a bacterial pathogen to assemble its own particulate vaccine capable of inducing cellular immunity. Sci. Rep..

[12] Peters V., Rehm B.H.A. (2006). In Vivo Enzyme Immobilization by Use of Engineered Polyhydroxyalkanoate Synthase. Appl. Environ. Microbiol..

[13] Parlane N.A., Grage K., Mifune J., Basaraba R.J., Wedlock N., Rehm B., Buddle B.M. (2012). Vaccines Displaying Mycobacterial Proteins on Biopolyester Beads Stimulate Cellular Immunity and Induce Protection against Tuberculosis. Clin. Vaccine Immunol..

[14] González-Miro M., Rodríguez-Noda L., Fariñas-Medina M., García-Rivera D., Vérez-Bencomo V., Rehm B.H. (2017). Self-assembled particulate PsaA as vaccine against *Streptococcus pneumoniae* infection. Heliyon.

[15] González-Miró M., Rodríguez-Noda L.M., Fariñas-Medina M., Cedré-Marrero B., Madariaga-Zarza S., Zayas-Vignier C., Hernández-Cedeño M., Kleffmann T., García-Rivera D., Vérez-Bencomo V. (2018). Bioengineered polyester beads co-displaying protein and carbohydrate-based antigens induce protective immunity against bacterial infection. Sci. Rep..

[16] Martínez-Donato G., Piniella B., Aguilar D., Olivera S., Pérez A., Castañedo Y., Alvarez-Lajonchere L., Dueñas-Carrera S., Lee J.W., Burr N. (2016). Protective T Cell and Antibody Immune Responses against Hepatitis C Virus Achieved Using a Biopolyester-Bead-Based Vaccine Delivery System. Clin. Vaccine Immunol..

[17] Moradali M.F., Rehm B.H.A. (2020). Bacterial biopolymers: From pathogenesis to advanced materials. Nat. Rev. Microbiol..

[18] Wibowo D., Jorritsma S.H., Gonzaga Z.J., Evert B., Chen S., Rehm B.H. (2021). Polymeric nanoparticle vaccines to combat emerging and pandemic threats. Biomaterials.

[19] Wu W., Huang J., Duan B., Traficante D.C., Hong H., Risech M., Lory S., Priebe G.P. (2012). Th17-stimulating Protein Vaccines Confer Protection againstPseudomonas aeruginosaPneumonia. Am. J. Respir. Crit. Care Med..

[20] Schaefers M.M., Duan B., Mizrahi B., Lu R., Reznor G., Kohane D.S., Priebe G.P. (2018). PLGA-encapsulation of the Pseudomonas aeruginosa PopB vaccine antigen improves Th17 responses and confers protection against experimental acute pneumonia. Vaccine.

[21] Baumann U., Mansouri E., Von Specht B.-U., Baumann U., Mansouri E., Von Specht B.-U. (2004). Recombinant OprF–OprI as a vaccine against Pseudomonas aeruginosa infections. Vaccine.

[22] Westritschnig K., Hochreiter R., Wallner G., Firbas C., Schwameis M., Jilma B. (2014). A randomized, placebo-controlled phase I study assessing the safety and immunogenicity of a Pseudomonas aeruginosa hybrid outer membrane protein OprF/I vaccine (IC43) in healthy volunteers. Hum. Vaccines Immunother..

[23] Cui Z., Han D., Sun X., Zhang M., Feng X., Sun C., Gu J., Tong C., Lei L., Han W. (2015). Mannose-modified chitosan microspheres enhance OprF-OprI-mediated protection of mice against Pseudomonas aeruginosa infection via induction of mucosal immunity. Appl. Microbiol. Biotechnol..

[24] Weimer E., Lu H., Kock N.D., Wozniak D.J., Mizel S.B. (2009). A Fusion Protein Vaccine Containing OprF Epitope 8, OprI, and Type A and B Flagellins Promotes Enhanced Clearance of Nonmucoid Pseudomonas aeruginosa. Infect. Immun..

[25] Rehm B., Boheim G., Tommassen J., Winkler U.K. (1994). Overexpression of algE in Escherichia coli: Subcellular localization, purification, and ion channel properties. J. Bacteriol..

[26] Rehm B.H., Grabert E., Hein J., Winkler U.K. (1994). Antibody response of rabbits and cystic fibrosis patients to an alginate-specific outer membrane protein of a mucoid strain of Pseudomonas aeruginosa. Microb. Pathog..

[27] Sambrook J., Fritsch E.F., Maniatis T. (1989). Molecular Cloning: A Laboratory Manual.

[28] Choi K.-H., Kumar A., Schweizer H.P. (2006). A 10-min method for preparation of highly electrocompetent Pseudomonas aeruginosa cells: Application for DNA fragment transfer between chromosomes and plasmid transformation. J. Microbiol. Methods.

[29] Spiekermann P., Rehm B., Kalscheuer R., Baumeister D., Steinbüchel A. (1999). A sensitive, viable-colony staining method using Nile red for direct screening of bacteria that accumulate polyhydroxyalkanoic acids and other lipid storage compounds. Arch. Microbiol..

[30] Evert B., Vezina B., Rehm B.H.A. (2020). Catalytically Active Bioseparation Resin Utilizing a Covalent Intermediate for Tagless Protein Purification. ACS Appl. Bio Mater..

[31] Brandl H., Gross R.A., Lenz R.W., Fuller R.C. (1988). Pseudomonas oleovorans as a Source of Poly(β-Hydroxyalkanoates) for Potential Applications as Biodegradable Polyesters. Appl. Environ. Microbiol..

[32] Rubio Reyes P., Parlane N.A., Wedlock D.N., Rehm B.H.A. (2016). Immunogencity of antigens from Mycobacterium tuberculosis self-assembled as particulate vaccines. Int. J. Med. Microbiol..

[33] Hay I.D., Hooks D.O., Rehm B.H.A. (2014). Use of Bacterial Polyhydroxyalkanoates in Protein Display Technologies. Hydrocarbon and Lipid Microbiology Protocols.

[34] Shevchenko A., Tomas H., Havlis J., Olsen J., Mann M.J. (2006). In-gel digestion for mass spectrometric characterization of proteins and proteomes. Nat. Protoc..

[35] Hatano K., Boisot S., DesJardins D., Wright D.C., Brisker J., Pier G.B., Hatano K., Boisot S., DesJardins D., Wright D.C. (1994). Immunogenic and antigenic properties of a heptavalent high-molecular-weight O-polysaccharide vaccine derived from Pseudomonas aeruginosa. Infect. Immun..

[36] Priebe G.P., Brinig M.M., Hatano K., Grout M., Coleman F.T., Pier G.B., Goldberg J.B. (2002). Construction and Characterization of a Live, Attenuated aroA Deletion Mutant of Pseudomonas aeruginosa as a Candidate Intranasal Vaccine. Infect. Immun..

[37] Choi K.-H., Gaynor J.B., White K.G., Lopez C., Bosio C.M., Karkhoff-Schweizer R.R., Schweizer H.P. (2005). A Tn 7-based broad-range bacterial cloning and expression system. Nat. Methods.

[38] Hoffmann N., Rehm B.H. (2004). Regulation of polyhydroxyalkanoate biosynthesis in Pseudomonas putida and Pseudomonas aeruginosa. FEMS Microbiol. Lett..

[39] Draper J.L., Rehm B.H. (2012). Engineering bacteria to manufacture functionalized polyester beads. Bioengineered.

[40] Pham T.H., Webb J., Rehm B.H.A. (2004). The role of polyhydroxyalkanoate biosynthesis by Pseudomonas aeruginosa in rhamnolipid and alginate production as well as stress tolerance and biofilm formation. Microbiology.

[41] Discola K.F., Förster A., Boulay F., Simorre J.-P., Attree I., Dessen A., Job V. (2014). Membrane and Chaperone Recognition by the Major Translocator Protein PopB of the Type III Secretion System of Pseudomonas aeruginosa. J. Biol. Chem..

[42] Dey S., Basu A., Datta S. (2012). Characterization of Molten Globule PopB in Absence and Presence of Its Chaperone PcrH. Protein J..

[43] Das S., Howlader D.R., Zheng Q., Ratnakaram S.S.K., Whittier S.K., Lu T., Keith J.D., Picking W.D., Birket S.E., Picking W.L. (2020). Development of a Broadly Protective, Self-Adjuvanting Subunit Vaccine to Prevent Infections by Pseudomonas aeruginosa. Front. Immunol..

[44] Grage K., Jahns A.C., Parlane N., Palanisamy R., Rasiah I.A., Atwood J.A., Rehm B. (2009). Bacterial Polyhydroxyalkanoate Granules: Biogenesis, Structure, and Potential Use as Nano-/Micro-Beads in Biotechnological and Biomedical Applications. Biomacromolecules.

[45] Huang M., Wang W. (2014). Factors affecting alum–protein interactions. Int. J. Pharm..

[46] Rehm B.H. (2017). Bioengineering towards self-assembly of particulate vaccines. Curr. Opin. Biotechnol..

[47] De Temmerman M.-L., Rejman J., Demeester J., Irvine D.J., Gander B., De Smedt S.C. (2011). Particulate vaccines: On the quest for optimal delivery and immune response. Drug Discov. Today.

[48] Ackerman A.L., Kyritsis C., Tampé R., Cresswell P. (2003). Early phagosomes in dendritic cells form a cellular compartment sufficient for cross presentation of exogenous antigens. Proc. Natl. Acad. Sci. USA.

[49] Foged C., Brodin B., Frokjaer S., Sundblad A. (2005). Particle size and surface charge affect particle uptake by human dendritic cells in an in vitro model. Int. J. Pharm..

[50] Limbach L.K., Li Y., Grass R.N., Brunner T.J., Hintermann M.A., Muller M., Gunther D., Stark W.J. (2005). Oxide nanoparticle uptake in human lung fibroblasts: Effects of particle size, agglomeration, and diffusion at low concentrations. Environ. Sci. Technol..

[51] Rehm B.H.A. (2003). Polyester synthases: Natural catalysts for plastics. Biochem. J..

[52] Zhang X., Yang F., Zou J., Wu W., Jing H., Gou Q., Li H., Gu J., Zou Q., Zhang J. (2018). Immunization with Pseudomonas aeruginosa outer membrane vesicles stimulates protective immunity in mice. Vaccine.

[53] Mizuno T., Kageyama M. (1978). Separation and Characterization of the Outer Membrane of Pseudomonas aeruginosa. J. Biochem..

[54] Koh A.Y., Priebe G.P., Ray C., Van Rooijen N., Pier G.B. (2009). Inescapable Need for Neutrophils as Mediators of Cellular Innate Immunity to Acute Pseudomonas aeruginosa Pneumonia. Infect. Immun..

[55] Di Giandomenico A., Rao J., Goldberg J.B. (2004). Oral Vaccination of BALB/c Mice with Salmonella enterica Serovar Typhimurium Expressing *Pseudomonas aeruginosa* O Antigen Promotes Increased Survival in an Acute Fatal Pneumonia Model. Infect. Immun..

[56] Pier G.B., Boyer D., Preston M., Coleman F.T., Llosa N., Mueschenborn-Koglin S., Theilacker C., Goldenberg H., Uchin J., Priebe G. (2004). Human Monoclonal Antibodies to Pseudomonas aeruginosaAlginate That Protect against Infection by Both Mucoid and Nonmucoid Strains. J. Immunol..

[57] Rehm B.H.A. (2010). Bacterial polymers: Biosynthesis, modifications and applications. Nat. Rev. Microbiol..

[58] Chen S., Sandford S., Kirman J.R., Rehm B.H.A. (2019). Innovative antigen carrier system for the development of tuberculosis vaccines. FASEB J..

[59] Rubio-Reyes P., Parlane N.A., Buddle B.M., Wedlock D.N., Rehm B.H.A. (2017). Immunological properties and protective efficacy of a single mycobacterial antigen displayed on polyhydroxybutyrate beads. Microb. Biotechnol..

[60] Parlane N.A., Wedlock D.N., Buddle B.M., Rehm B.H.A. (2009). Bacterial Polyester Inclusions Engineered to Display Vaccine Candidate Antigens for Use as a Novel Class of Safe and Efficient Vaccine Delivery Agents. Appl. Environ. Microbiol..

[61] Steinman L. (2007). A brief history of TH17, the first major revision in the TH1/TH2 hypothesis of T cell–mediated tissue damage. Nat. Med..

[62] Kolls J.K., Kanaly S.T., Ramsay A.J. (2003). Interleukin-17: An emerging role in lung inflammation. Am. J. Respir. Cell Mol. Biol..

[63] Chen K., McAleer J.P., Lin Y., Paterson D., Zheng M., Alcorn J.F., Weaver C., Kolls J.K. (2011). Th17 Cells Mediate Clade-Specific, Serotype-Independent Mucosal Immunity. Immunity.

[64] Komiyama Y., Nakae S., Matsuki T., Nambu A., Ishigame H., Kakuta S., Sudo K., Iwakura Y. (2006). IL-17 Plays an Important Role in the Development of Experimental Autoimmune Encephalomyelitis. J. Immunol..

[65] Pier G.B., Saunders J.M., Ames P., Edwards M.S., Auerbach H., Goldfarb J., Speert D.P., Hurwitch S. (1987). Opsonophagocytic Killing Antibody toPseudomonas aeruginosaMucoid Exopolysaccharide in Older Noncolonized Patients with Cystic Fibrosis. N. Engl. J. Med..

[66] Knirel Y.A. (1990). Polysaccharide Antigens of *Pseudomonas aeruginosa*. Crit. Rev. Microbiol..

[67] Fisher M.W., Devlin H.B., Gnabasik F.J. (1969). New immunotype schema for Pseudomonas aeruginosa based on protective antigens. J. Bacteriol..

[68] Moser C., Jensen P.O., Kobayashi O., Hougen H.P., Song Z., Rygaard J., Kharazmi A., Hby N. (2002). Improved outcome of chronic Pseudomonas aeruginosa lung infection is associated with induction of a Th1-dominated cytokine response. Clin. Exp. Immunol..

[69] Priebe G.P., Walsh R.L., Cederroth T.A., Kamei A., Coutinho-Sledge Y.S., Goldberg J.B., Pier G. (2008). IL-17 Is a Critical Component of Vaccine-Induced Protection against Lung Infection by Lipopolysaccharide-Heterologous Strains ofPseudomonas aeruginosa. J. Immunol..

[70] Yang F., Gu J., Yang L., Gao C., Jing H., Wang Y., Zeng H., Zou Q., Lv F., Zhang J. (2017). Protective Efficacy of the Trivalent Pseudomonas aeruginosa Vaccine Candidate PcrV-OprI-Hcp1 in Murine Pneumonia and Burn Models. Sci. Rep..

[71] Sainz-Mejías M., Jurado-Martín I., McClean S. (2020). Understanding *Pseudomonas aeruginosa*–Host Interactions: The Ongoing Quest for an Efficacious Vaccine. Cells.

[72] Wan C., Gao C., Xie Q., Wang Y., Cheng X., Fang Y., Liu Z., Zhang W., Zou Q., Lu G. (2021). Flagella hook protein FlgE is a novel vaccine candidate of Pseudomonas aeruginosa identified by a genomic approach. Vaccine.

[73] Bayes H.K., Bicknell S., MacGregor G., Evans T.J. (2014). T Helper Cell Subsets Specific for Pseudomonas aeruginosa in Healthy Individuals and Patients with Cystic Fibrosis. PLoS ONE.

[74] Roux D., Gaudry S., Dreyfuss D., El-Benna J., de Prost N., Denamur E., Saumon G., Ricard J.D. (2009). Candida albicans impairs macrophage function and facilitates Pseudomonas aeruginosa pneumonia in rat. Crit. Care Med..

