# Supplementary material for: A Pseudomonas aeruginosa-Derived Particulate Vaccine Protects against P. aeruginosa Infection
Source: Vaccines (Basel). 2021 Jul 20;9(7):803. doi: 10.3390/vaccines9070803 (PMC8309987; doi:10.3390/vaccines9070803)
Supplement: Supplementary file 1 [file vaccines-09-00803-s001.zip › vaccines-1295761-supplementary.pdf]

Table S1. Bacterial strains, plasmids and primers used in this study.

Strains, Plasmids and Primers	Relevant characteristics	References
**1. Bacterial strains**		
E. coli		
XL1-Blue	*recA1 endA1 gyrA96 thi-1 hsdR17 supE44 relA1 lac* [F′ *proAB lacI*^{q} *lacZ* Δ*M15* Tn*10* (Tet^{r})]	Stratagene
P. aeruginosa		
P. aeruginosa PAO1	Prototroph, non-mucoid	ATCC 15692
P. aeruginosa N13	Early CF clinical isolate, serotype O6, non-mucoid	[1]
PAO1Δ*phaC1ZC2*Δ*alg8*Δ*pelF*	PAO1Δ*phaC1ZC2*Δ*alg8* derivative with markerless, isogenic *pelF* deletion, triple mutant	[2]
PAO1Δ*phaC1ZC2*Δ*alg8*Δ*pelF*Δ*pslA*	PAO1Δ*phaC1ZC2*Δ*alg8*Δ*pelF* derivative with markerless, isogenic *pslA* deletion, quadruple mutant	This study
**2. Plasmids**		
pUC57	Cloning vector, ColE1 origin, Amp^{r}	Fermentas
pHERD20T	Amp^{r} Cb^{r}, pUCP20T P$_{lac}$ replaced with fragment of *araC*-PBAD cassette	[3]
pHERD20T-2	pHERD20T derivative were a 13 bp fragment of 5′ end of LacZ*α* is removed	[2]
pHERD20T-2_PhaC1	*P. aeruginosa* codon optimized PhaC1 fragment inserted into *Xba*I/*Hind*III site of pHERD20T-2	[2]
pHERD20T-2_Ag-PhaC1	*P. aeruginosa* codon optimized Ag fragment inserted into *Xba*I/*Nde*I sites of pHERD20T-2_PhaC1	[2]
pHERD20T-2_PhaC1-Ag	*P. aeruginosa* codon optimized Ag fragment inserted into *Xba*I/*Hind*III sites of pHERD20T-2_PhaC1	[2]
pUC57_XPopBS	pUC57 derivative containing *P. aeruginosa* codon optimized PopB fragment flanked by *Xba*I/*Sal*I sites	This study
pHERD20T-2_PopBAg-PhaC1	*P. aeruginosa* codon optimized PopB fragment from pUC57_XPopBS inserted into *Sal*I/*Xba*I sites of pHERD20T-2_Ag-PhaC1	This study
pHERD20T-2_PopB-PhaC1	*P. aeruginosa* codon optimized PopB fragment from pHERD20T-2_PopBAg-PhaC1, replacing Ag into *Xba*I/*Nde*I sites of pHERD20T-2_Ag-PhaC1	This study
pHERD20T-2_Ag-PhaC1-Ag	*P. aeruginosa* codon optimized Ag fragment inserted into *Xba*I/*Hind*III sites of pHERD20T-2_Ag-PhaC1	This study
pHERD20T-2_PhaC1-PopB	*P. aeruginosa* codon optimized PopB fragment from pUC57_XPopBS, replacing Ag into *Xho*I/*EcoR*I sites of pHERD20T-2_PhaC1-Ag	This study
pHERD20T-2_Ag-PhaC1-PopB	*P. aeruginosa* codon optimized PopB fragment from pUC57_XPopBS, replacing Ag into *Xho*I/*EcoR*I sites of pHERD20T-2_AgPhaC1-Ag	This study
pHERD20T-2_PhaC1-PopBAg	*P. aeruginosa* codon optimized PopBAg fragment from pHERD20T-2_PopBAg-PhaC1, replacing Ag into *Xho*I/*EcoR*I sites of pHERD20T-2_PhaC1-Ag	This study
**3. Primers**	**5′-3′**	
**Primer name**	**Sequence***	
XbaI popB_fwd	ATCCTCTAGAAGGAGATATACTTATGTTTGGTTGGATC	This study
NdeI popB_rev	ACTCATATGGCCACCGCCACTACCCGCTGCCGGTCGGCTG-GACAGGTTG	This study
Primer 5′ (XhoI)	AAAAA CTCGAGTTTGGTTGGATCAGTGCAATAGCTTCGATC	This study
Primer 3′ (EcoRI)	AAAAAAGAATTCTCAGATCGCTGCCGGTCGGCTGGACAGGTTG	This study
XhoI-2	ACAAACTCGAGTTTGGTTGGATCAGTGCAATAGCTTCG	This study
EcoRI-2	AAAAAAGAATTCTCACTTGCGGCTCGCCTTCTCC	This study

Amp^{r}, ampicillin resistance; Gm^{r}, gentamicin resistance; Tet^{r}, tetracycline resistance; Cm^{r} chloramphenicol resistance.
*Restriction sites are underlined.

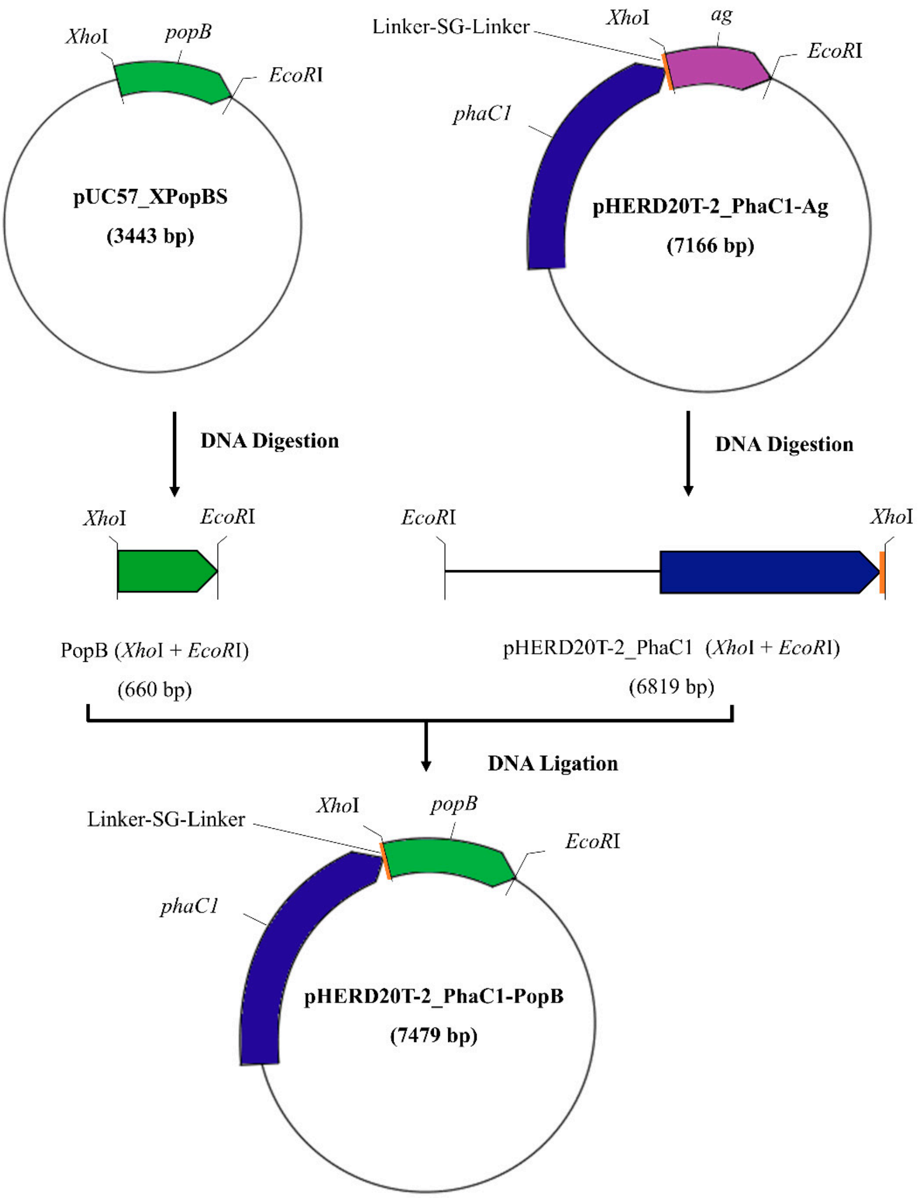

Figure S1. Strategy for the construction of pHERD20T-2_PhaC1-PopB for PHA-PopB bead vaccine production. The *Pseudomonas* codon optimized PopB fragment was isolated from pUC57_XPopBS by PCR amplification using primers Primer 5′ (XhoI) and Primer 3′ (EcoRI), followed by recovery of DNA fragments using agarose gel electrophoresis and gel purification. The recovered PopB fragment was digested using restriction enzymes *Xho*I and *EcoR*I, and subjected to purification. The PopB fragment was ligated using T4 DNA ligase into the linearized vector pHERD20T-2_PhaC1 which was generated by restriction enzyme digest with *Xho*I and *EcoR*I, generating the final plasmid pHERD20T-2_PhaC1-PopB.

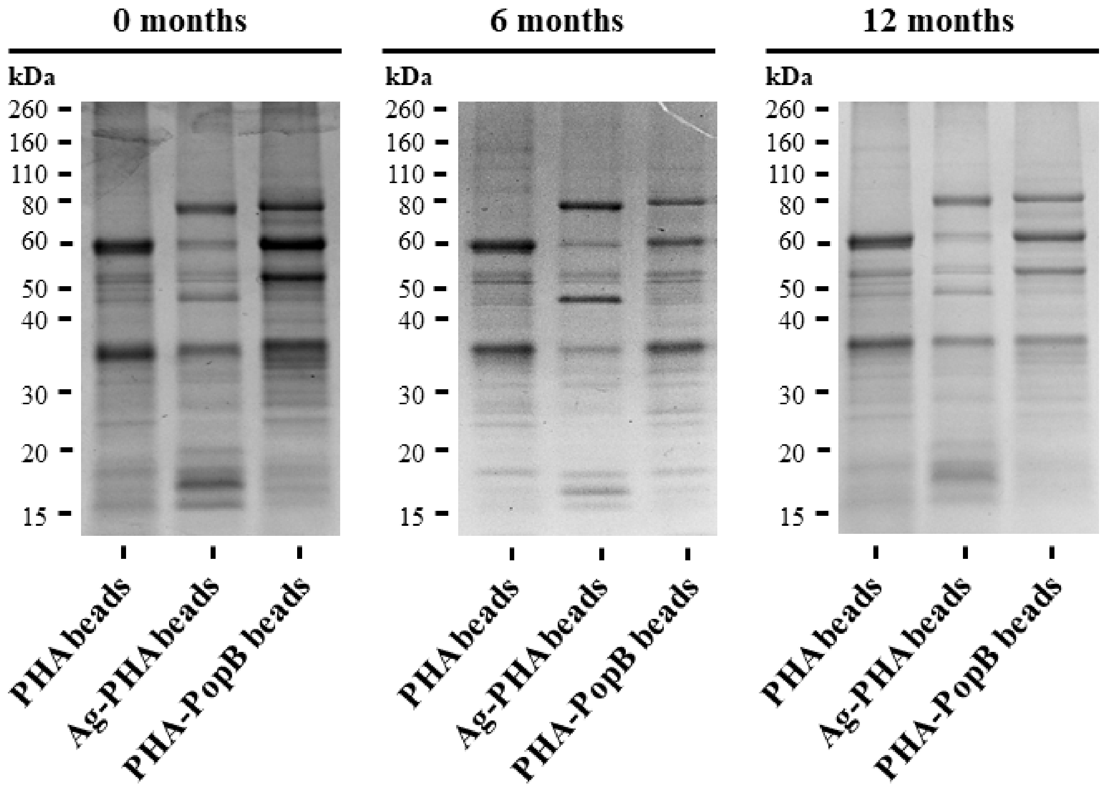

Figure S2. Stability study. Protein profiles of PHA bead formulation were assessed after storage for 0, 6 and 12 months at 4°C by SDS-PAGE. The gel was stained with Coomassie Blue. PHA (63.2 kDa), Ag-PHA (77.7 kDa), and PHA-PopB (86.4 kDa) fusion proteins along with the HCPs were isolated from *P. aeruginosa* PAO1ΔCΔ8ΔF strain containing the respective plasmids.

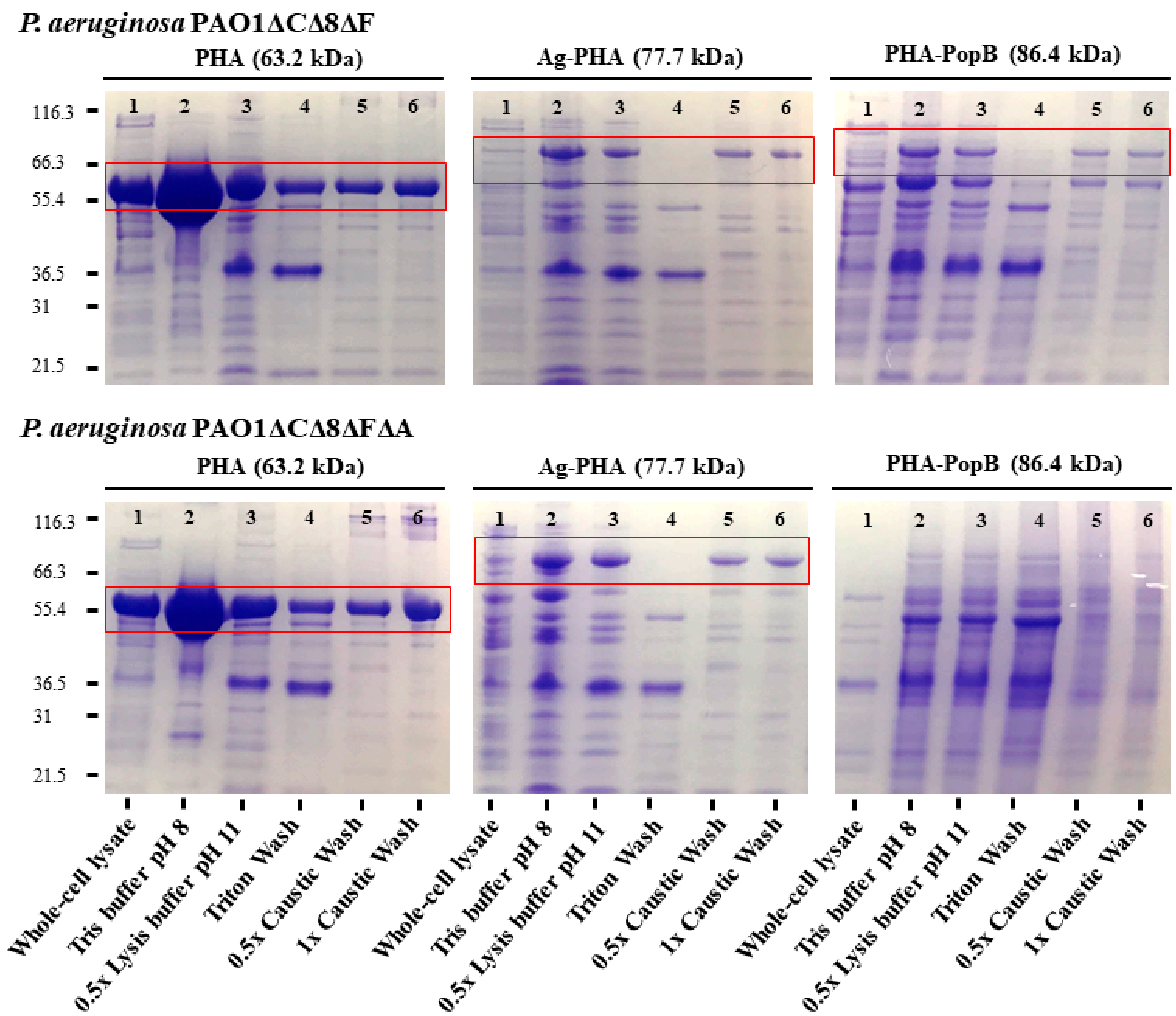

Figure S3. Target fusion protein production in *P. aeruginosa* PAO1ΔCΔ8ΔF and PAO1ΔCΔ8ΔFΔA production strains containing the respective plasmids for the production of PHA, Ag-PHA and PHA-PopB, respectively. Target fusion proteins are in red boxes. Lane 1, whole-cell lysate; lanes 2-6, different washing buffers that were tested for purification.

Table S2. Sequences of OprF, OprI and AlgE.

Antigens	Sequences
OprF	NATAEGRAINRRVE
OprI	SSHSKETEARLTATEDAAARAQARADEAYRKADEALGAAQKAQQTADEANERALRMLEKASRK
AlgE	HLRRPGEEVNLTTTTVDDRRIATGKQ

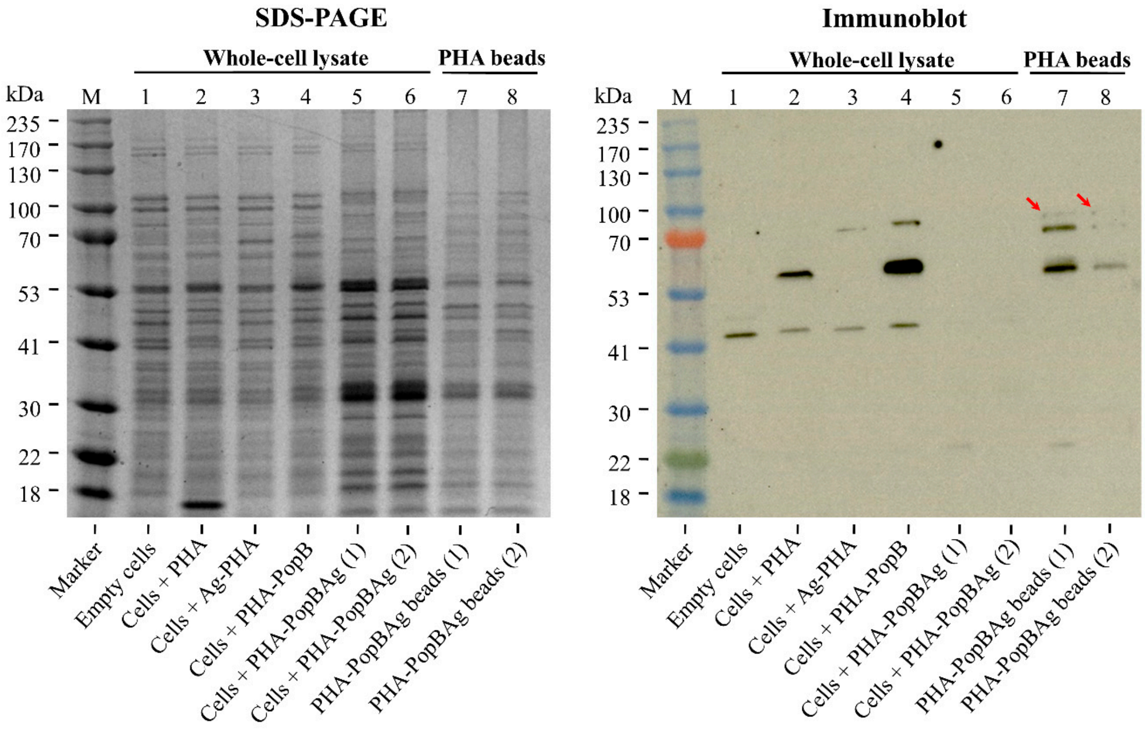

Figure S4. Immunoblot for detection of target fusion proteins using anti-PHA synthase antibodies. Two sets of PHA-PopBAg beads were produced to assess the PHA-PopBAg fusion protein production using SDS-PAGE and immunoblot. PHA-PopBAg fusion proteins were visible only in western blot and in the isolated PHA-PopBAg beads (red arrows) but not in the corresponding whole-cells. .

Table S3. Particle size and zeta-potential measurements of the PHA bead vaccines.

PHB beads	Ave. Size (μm)	Std. dev. (Size)	Ave. PDI	Ave. Zeta Potential (mV)	Std. dev. (Zeta Potential)
PHA beads	0.556	0.045	0.42	-12.27	0.351
Ag-PHA beads	0.323	0.015	0.28	-13.60	0.721
PHA-PopB beads	0.238	0.034	0.50	-13.83	0.839
Alhydrogel	1.720	0.228	0.31	11.33	1.528
PHA beads + Alhydrogel	3.373	0.139	0.55	-9.14	0.230
Ag-PHA beads + Alhydrogel	2.207	0.181	0.40	-8.39	0.396
PHA-PopB beads + Alhydrogel	4.168	0.363	0.53	-8.80	0.425

Ave. (Average); Std. dev. (Standard deviation); PDI (Polydispersity index).

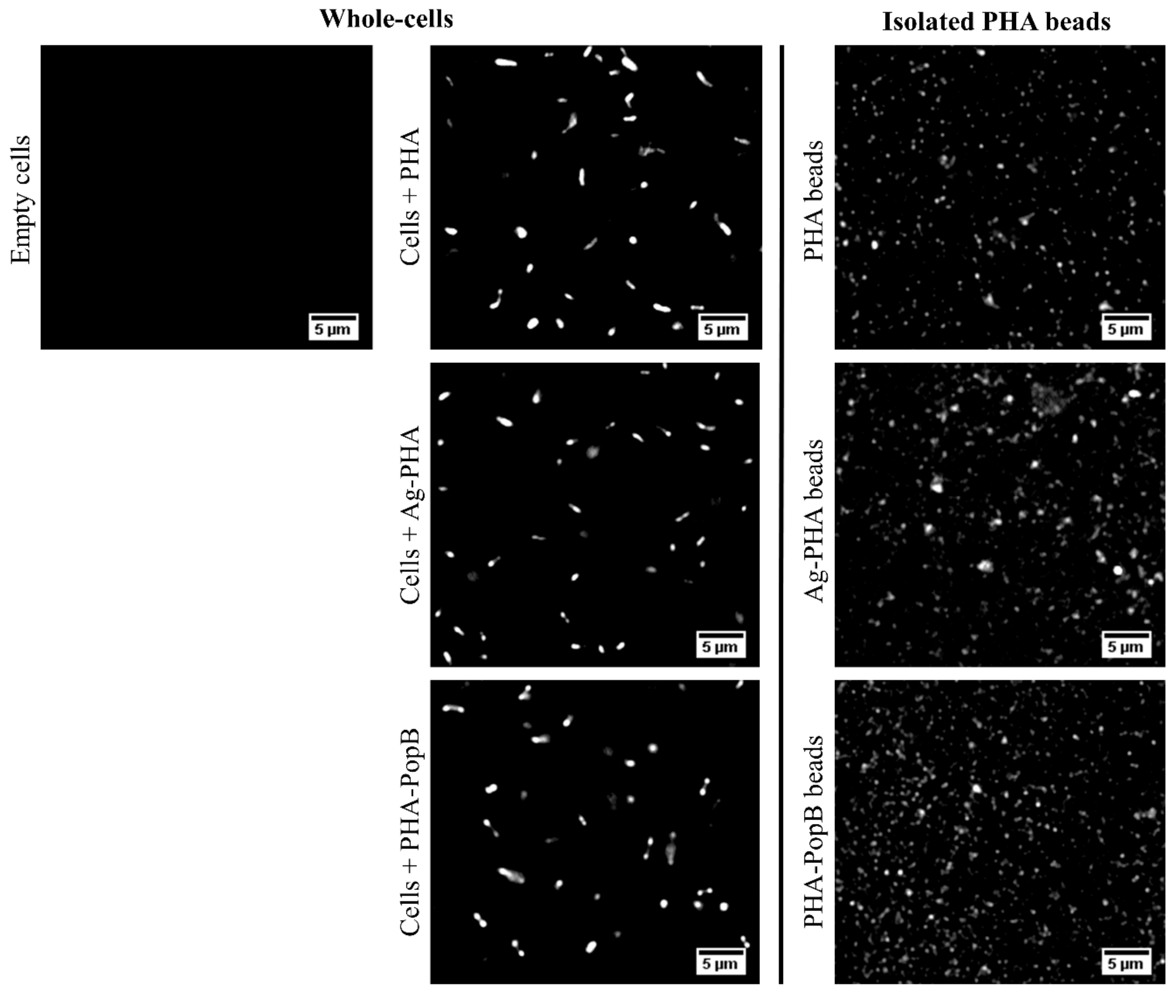

Figure S5. Fluorescence microscopy images of whole-cells and PHA beads produced in bioengineered *P. aeruginosa* PAO1ΔCΔ8ΔF harbouring various fusion protein-encoding pHERD20T-2 expression vectors stained with Nile-red. Fluorescence was detected in whole-cells containing respective plasmids, and the isolated PHA, Ag-PHA and PHA-PopB beads. No fluorescence was detected in the negative control empty cells (*P. aeruginosa* PAO1ΔCΔ8ΔF).

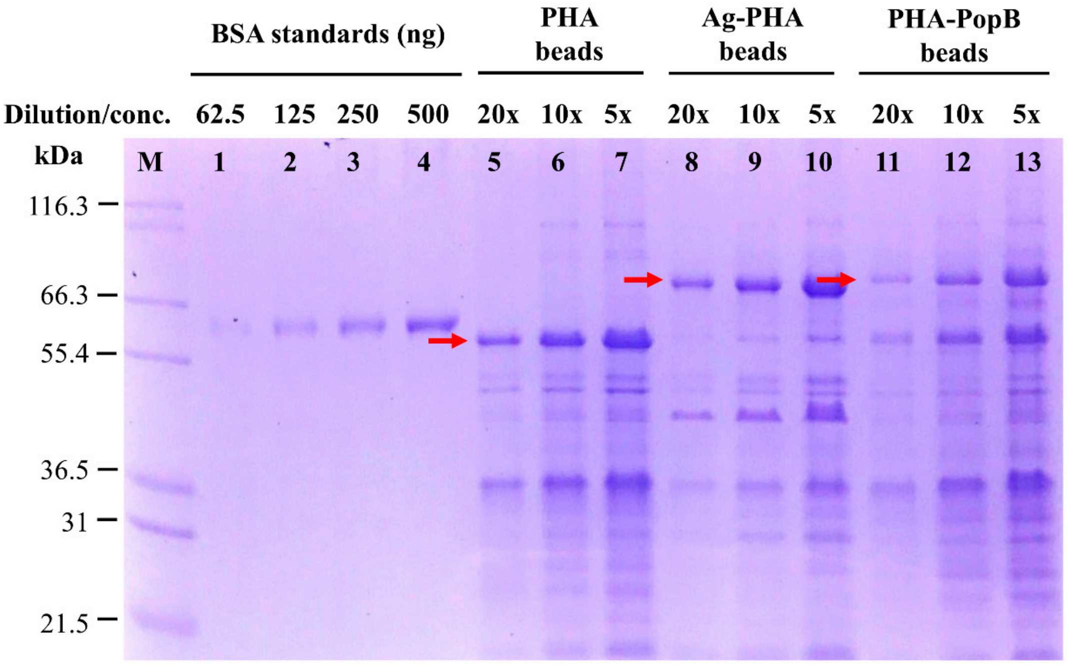

Figure S6. Protein quantification of the purified PHA bead vaccines by densitometry. Different amounts of BSA standard ranging between 62.5 and 500 ng were loaded on Bis-Tris gel to generate a standard curve, used to determine the antigen concentrations. The image analysed using the Image J version 1.52a (National Institute of Health). Target fusion proteins are in red arrows.

Table S4. Mass Spectrometry (MS) analysis for identification of PHA synthase and PHA synthase fusion proteins.

Protein Sequence	Protein sequence coverage and the confirmed fragments
**PHA (PhaC1, MW: 63.2 kDa)**<br>1 MSQKNNNELPKQAAENTLNLNPVIGIRGKDLLTSARMVLLQAVRQPLHSA<br>51 RHVAHFSLELKNVLLGQSELRPGDDDRRFSDPAWSQNPLYKRYMQTYLAW<br>101 RKELHSWISHSDLSPQDISRGQFVINLLTEAMSPTNSLSNPAAVKRFFET<br>151 GGKSLLDGLGHLAKDLVNNGGMPSQVDMDAFEVGKNLATTEGAVVFRNDV<br>201 LELIQYRPITESVHERPLLVVPPQINKFYVFDLSPDKSLARFCLRNGVQT<br>251 FIVSWRNPTKSQREWGLTTYIEALKEAIEVVLSITGSKDLNLLGACSGGI<br>301 TTATLVGHYVASGEKKVNAFTQLVSVLDFELNTQVALFADEKTLEAAKRR<br>351 SYQSGVLEGKDMAKVFAWMRPNDLIWNYWVNNYLLGNQPPAFDILYWNND<br>401 TTRLPAALHGEFVELFKSNPLNRPGALEVSGTPIDLKQVTCDFYCVAGLN<br>451 DHITPWESCYKSARLLGGKCEFILSNSGHIQSILNPPGNPKARFMTNPEL<br>501 PAEPKAWLEQAGKHADSWWLHWQQWLAERSGKTRKAPASLGNKTYPAGEA<br>551 APGTYVHER	73%<br>N5- R27, F79-K91, Y93-R197, F228-R241, N246-R256, E264-K342, S351-K461, C470-K491,H514-R529, T544-R559
**Ag-PHA (Ag-PhaC1, MW: 77.7 kDa)**<br>1 MHLRRPGEEVNLTTTTVDDRRIATGKQNATAEGRAINRRVENATAEGRAI<br>51 NRRVENATAEGRAINRRVESSHSKETEARLTATEDAAARAQARADEAYRK<br>101 ADEALGAAQKAQQTADEANERALRMLEKASRKMSQKNNNELPKQAAENTL<br>151 NLNPVIGIRGKDLLTSARMVLLQAVRQPLHSARHVAHFSLELKNVLLGQS<br>201 ELRPGDDDRRFSDPAWSQNPLYKRYMQTYLAWRKELHSWISHSDLSPQDI<br>251 SRGQFVINLLTEAMSPTNSLSNPAAVKRFFETGGKSLLDGLGHLAKDLVN<br>301 NGGMPSQVDMDAFEVGKNLATTEGAVVFRNDVLELIQYRPITESVHERPL<br>351 LVVPPQINKFYVFDLSPDKSLARFCLRNGVQTFIVSWRNPTKSQREWGLT<br>401 TYIEALKEAIEVVLSITGSKDLNLLGACSGGITTATLVGHYVASGEKKVN<br>451 AFTQLVSVLDFELNTQVALFADEKTLEAAKRRSYQSGVLEGKDMAKVFAW<br>501 MRPNDLIWNYWVNNYLLGNQPPAFDILYWNNDTTRLPAALHGEFVELFKS<br>551 NPLNRPGALEVSGTPIDLKQVTCDFYCVAGLNDHITPWESCYKSARLLGG<br>601 KCEFILSNSGHIQSILNPPGNPKARFMTNPELPAEPKAWLEQAGKHADSW<br>651 WLHWQQWLAERSGKTRKAPASLGNKTYPAGEAAPGTYVHER	62%<br>R4-R20, Q144-R159, F211-K223, Y225-R278, D297-R329, L333-K369, N378-R388, E396-K474, R482-K593, C602-K623, H646-R661, T676-R691
**PHA-PopB (PhaC1-PopB, MW: 86.4 kDa)**<br>1 MSQKNNNELPKQAAENTLNLNPVIGIRGKDLLTSARMVLLQAVRQPLHSA<br>51 RHVAHFSLELKNVLLGQSELRPGDDDRRFSDPAWSQNPLYKRYMQTYLAW<br>101 RKELHSWISHSDLSPQDISRGQFVINLLTEAMSPTNSLSNPAAVKRFFET<br>151 GGKSLLDGLGHLAKDLVNNGGMPSQVDMDAFEVGKNLATTEGAVVFRNDV<br>201 LELIQYRPITESVHERPLLVVPPQINKFYVFDLSPDKSLARFCLRNGVQT<br>251 FIVSWRNPTKSQREWGLTTYIEALKEAIEVVLSITGSKDLNLLGACSGGI<br>301 TTATLVGHYVASGEKKVNAFTQLVSVLDFELNTQVALFADEKTLEAAKRR<br>351 SYQSGVLEGKDMAKVFAWMRPNDLIWNYWVNNYLLGNQPPAFDILYWNND<br>401 TTRLPAALHGEFVELFKSNPLNRPGALEVSGTPIDLKQVTCDFYCVAGLN<br>451 DHITPWESCYKSARLLGGKCEFILSNSGHIQSILNPPGNPKARFMTNPEL<br>501 PAEPKAWLEQAGKHADSWWLHWQQWLAERSGKTRKAPASLGNKTYPAGEA<br>551 APGTYVHERFGWISAIASIIVGAIMVATGVGAAAGALMIAGGVMGVVSQ<br>601 SVQQAAADGLISKEVMEKLGPALMGIEMAVALLAAVVSFGGSAVGGLARL<br>651 GAKIGGKAAEMTASLASKVADLGGKFGSLAGQSLSHSLKLGVQVSDLTLD	61%<br>Q12-R27, F79-K91, Y93-R146, D165-K237, N246-R256, E264-K342, V365-K461, C470-K491, H514-R529, T544-R559, A658-K668, F676-K716, A729-R740, M748-R758

701 VANGAAQATHSGFQAKAANRQADVQESRADLTTLQGVIERLKEELSRMLE
751 AFQEIMERIFAMLQAKGETLHNLSSRPAAI

*Confirmed sequences are in red colour. MW (Molecular weight). Yellow highlights are the respective Ag and PopB sequences.

Table S5. Identification of the nine PHA bead associated HCPs by mass spectrometry.

Band[a]	Rank[b]	Database hits
I	1	RecName: Full=Outer membrane porin F; Flags: Precursor
	2	RecName: Full=Outer membrane protein assembly factor BamD; Flags: Precursor
	3	2-alkenal reductase [Pseudomonas aeruginosa]
	4	hypothetical protein [Pseudomonas aeruginosa]
	5	efflux RND transporter periplasmic adaptor subunit [Pseudomonas aeruginosa]
	6	RecName: Full=UDP-N-acetylglucosamine--N-acetylmuramyl-(pentapeptide) pyrophosphoryl-undecaprenol N-acetylglucosamine transferase; AltName: Full=Undecaprenyl-PP-MurNAc-pentapeptide-UDPGlcNAc GlcN...
II	1	alpha/beta hydrolase [Pseudomonas aeruginosa]
	2	MULTISPECIES: type VI secretion-associated lipoprotein TagQ [Pseudomonas]
	3	RecName: Full=ATP synthase gamma chain; AltName: Full=ATP synthase F1 sector gamma subunit; AltName: Full=F-ATPase gamma subunit
	4	ABC transporter permease, partial [Pseudomonas aeruginosa]
	5	universal stress protein, partial [Pseudomonas aeruginosa]
	6	acyl dehydratase [Pseudomonas aeruginosa]
	7	hypothetical protein [Pseudomonas aeruginosa]
III	1	peptidylprolyl isomerase [Pseudomonas aeruginosa]
	2	RecName: Full=30S ribosomal protein S2
	3	ParA family protein [Pseudomonas aeruginosa]
	4	RecName: Full=50S ribosomal protein L1
IV	1	RecName: Full=Peptidoglycan-associated lipoprotein; Flags: Precursor
	2	nuclear transport factor 2 family protein [Pseudomonas aeruginosa]
	3	RecName: Full=Phosphopantetheine adenylyltransferase; AltName: Full=Dephospho-CoA pyrophosphorylase; AltName: Full=Pantetheine-phosphate adenylyltransferase; Short=PPAT
	4	3-hydroxyacyl-[acyl-carrier-protein] dehydratase FabZ [Pseudomonas aeruginosa]
V	1	class II poly(R)-hydroxyalkanoic acid synthase [Pseudomonas aeruginosa]
	2	molecular chaperone GroEL [Pseudomonas aeruginosa]
	3	MULTISPECIES: ShlB/FhaC/HecB family hemolysin secretion/activation protein [Pseudomonas]
	4	MULTISPECIES: hypothetical protein [Pseudomonas]
	5	methyl-accepting chemotaxis protein [Pseudomonas aeruginosa]
	6	methyl-accepting chemotaxis protein [Pseudomonas aeruginosa]
	7	RecName: Full=Glutamine synthetase; Short=GS; AltName: Full=Glutamate--ammonia ligase; AltName: Full=Glutamine synthetase I beta; Short=GSI beta
	8	MULTISPECIES: ATP-dependent RNA helicase RhlB [Pseudomonas]
VI	1	RecName: Full=Peptidoglycan-associated lipoprotein; Flags: Precursor
	2	RecName: Full=30S ribosomal protein S7
	3	3-hydroxyacyl-[acyl-carrier-protein] dehydratase FabZ [Pseudomonas aeruginosa]
VII	1	class II poly(R)-hydroxyalkanoic acid synthase [Pseudomonas aeruginosa]
	2	hypothetical protein PA2540 [Pseudomonas aeruginosa PAO1]
	3	hypothetical protein [Pseudomonas aeruginosa]
	4	ShlB/FhaC/HecB family hemolysin secretion/activation protein [Pseudomonas aeruginosa]
VIII	1	RecName: Full=Outer membrane porin F; Flags: Precursor
	2	RecName: Full=Outer membrane protein assembly factor BamD; Flags: Precursor
	3	MULTISPECIES: glycoside hydrolase family 43 [Pseudomonas]
	4	AraC family transcriptional regulator [Pseudomonas aeruginosa]
	5	serine/threonine protein kinase [Pseudomonas aeruginosa]
	6	alpha/beta hydrolase [Pseudomonas aeruginosa]
	7	RecName: Full=Dihydroorotate dehydrogenase (quinone); AltName: Full=DHOdehase; Short=DHOD; Short=DHODase; AltName: Full=Dihydroorotate oxidase
IX	1	rhamnosyltransferase [Pseudomonas aeruginosa PAO1]
	2	cell division protein ZipA [Pseudomonas aeruginosa]

3	imelysin [Pseudomonas aeruginosa]
4	RNA-directed DNA polymerase [Pseudomonas aeruginosa]
5	alpha/beta hydrolase [Pseudomonas aeruginosa]
6	AraC family transcriptional regulator [Pseudomonas aeruginosa]
7	biofilm formation protein PslC [Pseudomonas aeruginosa PAO1]

[a] Protein bands identified on SDS-PAGE. [b] Peptide filter (PeptideProphet ≥ 0.995), protein filter (UniquePepties ≥ 6) and filtered by approximate molecular weight. The rank is based on the number of 'unique peptides'; the higher the number of 'unique peptides', the higher is the ranking.

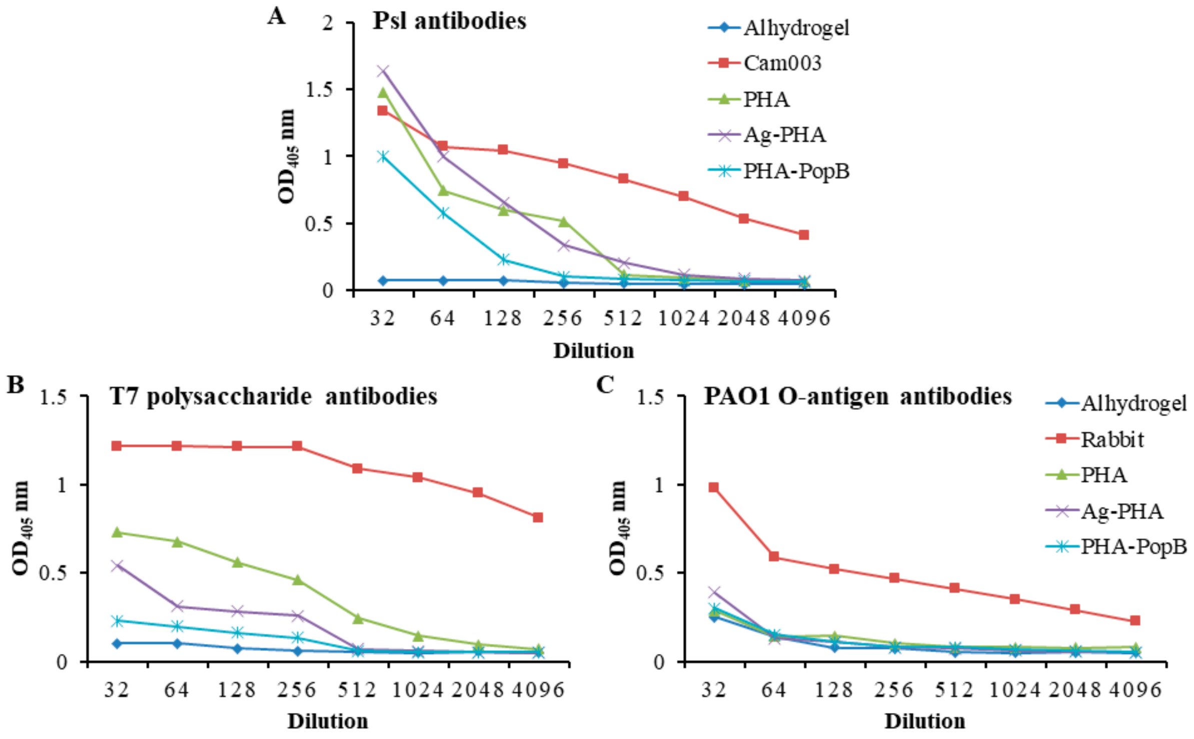

Figure S7. Anti-Psl and anti-LPS O-antigen polysaccharide responses in mice vaccinated with various PHA beads. ELISA plates were coated with the various polysaccharides and binding of antibodies in pooled sera was assessed. **(A)** Anti-Psl antibodies. Human anti-Psl mAb (Cam-003) is the positive control. **(B)** Anti-T7 LPS antibodies. Rabbit antisera to live-attenuated *P. aeruginosa* PAO1ΔaroA is the positive control. **(C)** Anti-PAO1 O-antigen polysaccharide antibodies. Rabbit antisera to live-attenuated *P. aeruginosa* PAO1ΔaroA is the positive control.

References

[1] Koh AY, Priebe GP, Ray C, Van Rooijen N, Pier GB. Inescapable need for neutrophils as mediators of cellular innate immunity to acute *Pseudomonas aeruginosa* pneumonia. Infection and immunity. 2009;77:5300-10.

[2] Lee JW, Parlane NA, Wedlock DN, Rehm BHA. Bioengineering a bacterial pathogen to assemble its own particulate vaccine capable of inducing cellular immunity. Sci Rep. 2017;7:41607.

[3] Qiu D, Damron FH, Mima T, Schweizer HP, Hongwei DY. PBAD-based shuttle vectors for functional analysis of toxic and highly regulated genes in *Pseudomonas* and *Burkholderia* spp. and other bacteria. Applied and environmental microbiology. 2008;74:7422-6.
